# Characteristic Aspects of Additive Manufacturing Security From Security Awareness Perspectives

**DOI:** 10.1109/access.2019.2931738

**Published:** 2019

**Authors:** LYNNE M. G. GRAVES, JOSHUA LUBELL, WAYNE KING, MARK YAMPOLSKIY

**Affiliations:** 1 Department of Computer Science, University of South Alabama, Mobile, AL 36688, USA; 2 National Institute of Standards and Technology, Gaithersburg, MD 20899, USA; 3 Lawrence Livermore National Laboratory, Livermore, CA 94550, USA; 4 Department of Computer Science and Software Engineering, Auburn University, Auburn, AL 36849, USA

**Keywords:** AM security, additive manufacturing, 3D printing, subtractive manufacturing, security, safety

## Abstract

Additive manufacturing (AM) is expected to become an established manufacturing technology in the near future. The growing penetration of AM at manufacturers across the world and the dependence of this technology on computerization have already raised security concerns, some of which have been proven experimentally. In this paper, we analyze the AM Security from three awareness perspectives: exposure to an attack, evaluation of the system, and potential liability for a successful attack. For each of these perspectives, we first introduce the conceptual background and then provide the analysis of its applicability to AM, highlighting its distinctiveness from closely related subtractive manufacturing (SM). Our analysis shows that, while there is a certain overlap between AM and SM security, the AM requires a separate and unique perspective and approach undertaken by experts with relevant domain expertise.

## I. INTRODUCTION

Additive Manufacturing (AM) is a growing manufacturing technology. Gartner has predicted that within the next 5 to 10 years 3D printing in manufacturing operations will have reached the “plateau of productivity” while 3D printing service bureaus have already reached that stage and are less than 2 years from becoming mainstream and widely adopted [1]. According to the Wohler’s Report 2018, AM products and services are expected to reach $11.7 billion in 2019 sales and $27.3 billion by 2023, a 372 % increase over actual sales in 2017 [2].

As the technology continues to mature, AM parts are being incorporated increasingly into security-sensitive and safety-critical products. The United States Army has been using additive manufacturing for M1 Abrams tank repair parts [3] and has demonstrated an additively manufactured grenade launcher using a 3D printed grenade [4]. The United States Navy has installed an additively manufactured drain strainer in an aircraft carrier’s steam system [5] and flown additively manufactured metal flight critical parts in naval aircraft [6], [7]. The United States Air Force has replaced aluminum F-22 Raptor Stealth Fighter brackets with additively manufactured titanium pieces [8]. The titanium brackets are expected to last longer due to corrosion resistance and have a shorter notice production lead-time, providing increased aircraft readiness. The Air Force has plans to incorporate at least five more metallic AM parts in the F-22 resulting in a significant reduction in maintenance down-time.

Non-military implementations of AM include the General Electric fuel injection nozzle used in the Airbus A320neo Leading Edge Aviation Propulsion jet engine [9], the Super-Draco Engine Chamber in the Space X Dragon Version 2 vehicle [10], the BE-7 Engine used in the Blue Origin Blue Moon Lunar Lander [11], [12] and brake cooling ducts for the McLaren Formula 1 MCL32 racing car [13], [14].

With such broad application areas and a high level of computerization, AM is considered a likely target for malicious actors who could, for example, sabotage the printed product or the AM equipment [15]. While AM is subject to the same attack vectors as other cyber-physical systems (CPS) [16], it also faces unique challenges. One approach to analyzing AM security is to consider it from security awareness perspectives. From these perspectives, the system owner first must become aware that there is a viable risk, then they must engage in risk assessments and contemplate mitigations, and, lastly, the decision maker must weigh potential liability exposure when determining which, if any, security measures to implement.

**FIGURE 1.**
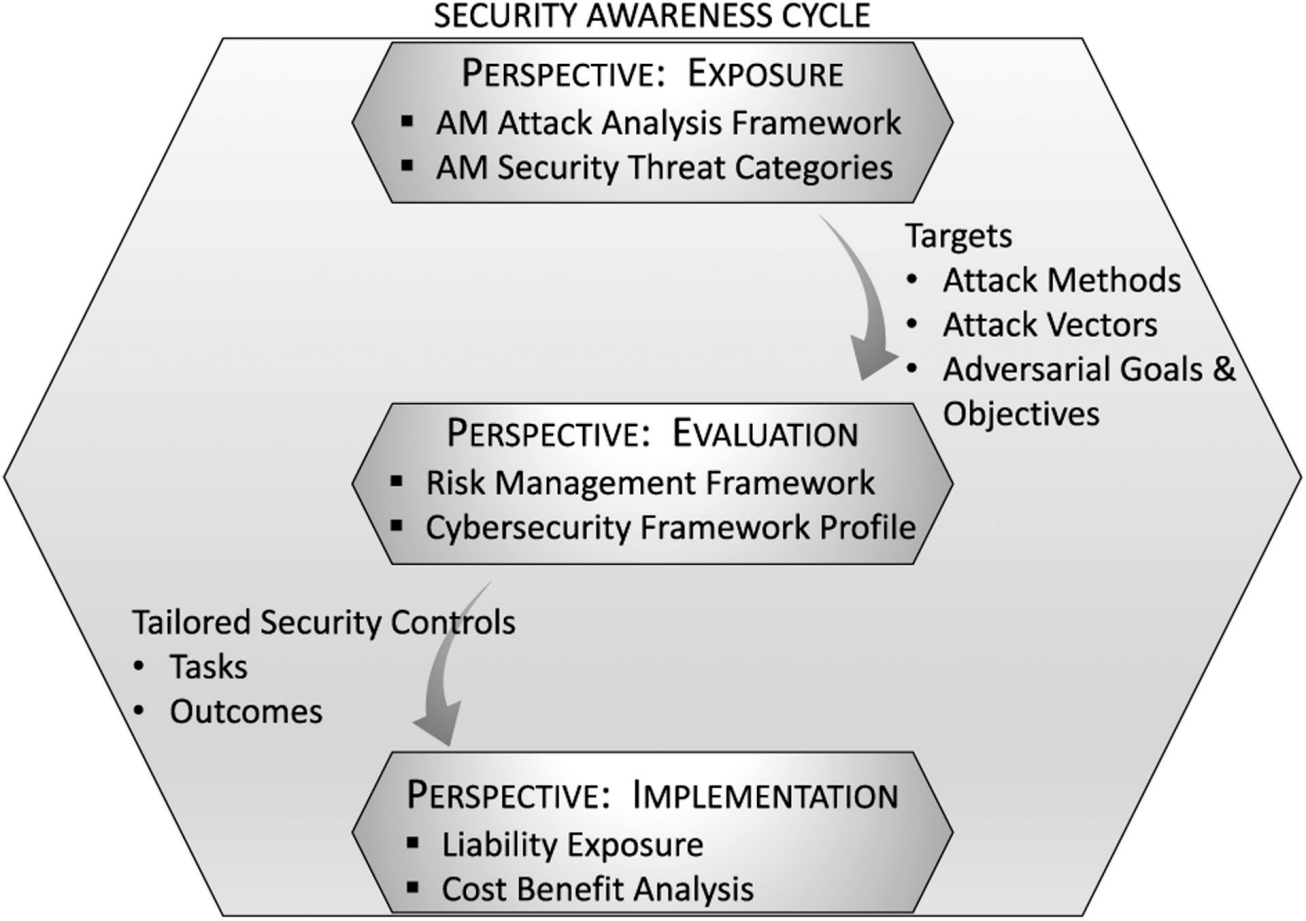
AM security awareness cycle.

Such an approach is common for implementers with newly adopted technologies where implementation and functionality have developed independently of security. As often is the case, the new technology inherits the security of similar processes. Here, AM has inherited the security related to traditional Subtractive Manufacturing (SM) using Computer Numerical Controlled (CNC) machines. By analyzing AM security along a security awareness cycle, we identify areas in which AM security is unique and differs from SM security. Fig. 1 summarizes the different security awareness perspectives and their interrelatedness in the awareness cycle.

We first outline the different security decision making perspectives in Section II. In Section III, we provide a brief AM background and then discuss attack vectors, attack methods, and targets applicable to AM. We then examine AM security with respect to system evaluation in Section IV. In Section V, we consider potential liability exposure which impacts mitigation implementation decisions. In Section VI, we summarize our analyses from the prior sections. Finally, in Section VII, we present our conclusions and highlight the need for further AM security research. Throughout this paper, we provide reference citations within tables and figures. The reference citations use a mnemonic concatenation of the author surname, publication year, and first word of the title. Table 1 provides a summary of the mnemonics and full citations used in this paper.

## II. SECURITY PERSPECTIVES

Theoretically, security should be incorporated from the beginning stages of a new cyber-dependent technology [17]–[20]. However, there is often a different security awareness life cycle demonstrated when adopting new technology. The initial emphasis on new technology is functionality, not security. With limited resources and time-to-market pressure, designers and implementers focus on proofs of concept and expanding functionality. Furthermore, when a technology is perceived to be an expansion of a current capability, such as manufacturing in this case, the new technology inherits the existing security posture. As such, the implementers must first be made aware of security threats unique to the new technology. Awareness can come through a real-life threat occurrence or a persuasive analysis such as that presented in Section III.

Once there is an awareness that new technology faces unique security challenges, it becomes necessary to assess the system and its associated risk. In the second awareness phase, the implementers evaluate their technology security, both comparing it to existing technology with known security risks and evaluating it for new, differentiating risks. Effective tools and frameworks for system security evaluation and mitigation strategy formulations include the National Institute for Standards and Technology (NIST) Risk Management Framework (RMF) [21] and the NIST Cybersecurity Framework [22]. Section IV discusses unique considerations for AM.

Following an awareness of threats and an assessment of risks, the technology adopter must weigh which security steps to pursue. The security recommendations can impact the functionality of the system as well as the operational cost. It is not usually feasible, even in the cyber-security domain, to implement all security recommendations completely; the recommendations are prioritized on a cost-benefit analysis which necessarily includes liability exposure. Thus, in the last phase of this security awareness cycle, the implementers must make security integration decisions which reflect a cost-benefit analysis in consideration of liability exposure. Liability exposure is considered in Section V.

## III. PERSPECTIVE I: EXPOSURE

In this section we first outline the background necessary to understand the exposure of AM^1^ to various attacks. Then we provide an exposure analysis, indicating whether the identified elements are unique to AM or also applicable to Subtractive Manufacturing (SM) using computer numerical control (CNC) machines. Our analysis is limited to SM^2^ and does not consider other manufacturing technologies such as injection molding or casting and their respective cyber-security issues in order to provide a concise analysis within a reasonable length. SM has been selected for this analysis as it is more readily comparable to AM with regards cyber-security issues. Consideration of AM cyber-security issues in the context of other manufacturing technologies is of interest for future research.

### A. BACKGROUND

Additive Manufacturing (AM) is not an isolated process; it is embedded in a complex interaction of manual and automated workflows in which various dependencies — including physical and informational — can be defined. AM workflows may vary drastically based on the AM process employed,^3^ source materials used, and whether manufacturing is performed by end users or is provided as a service.^4^

**FIGURE 2.**
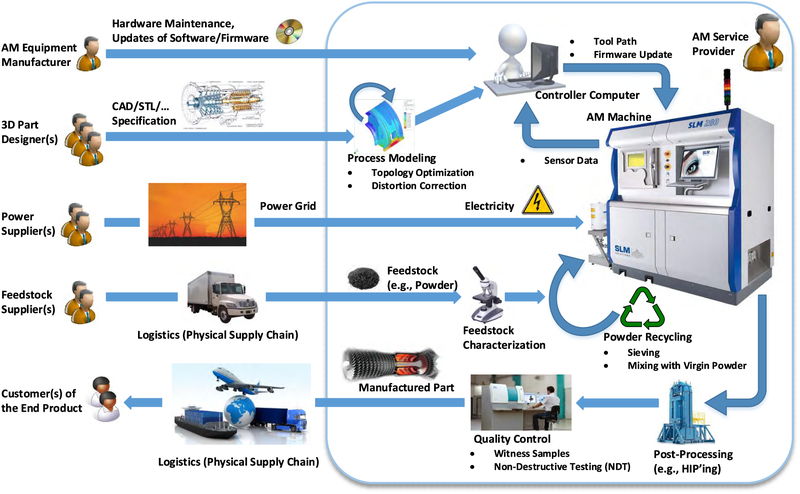
Additive manufacturing workflow, from design to supply to production [23].

Fig. 2 depicts a representative workflow when metal AM^5^ is provided as a service. The workflow components include several actors, computerized systems, software applications, data transfer mechanisms, and physical item transportation [23]. Not all elements in the workflow are located in the AM service provider’s *controlled environment* (indicated by the rectangular area in Fig. 2). Multiple actors, most of which represent enterprises, are involved in AM and provide or utilize the various services. Such a complex workflow is susceptible to a broad range of attacks.

**FIGURE 3.**
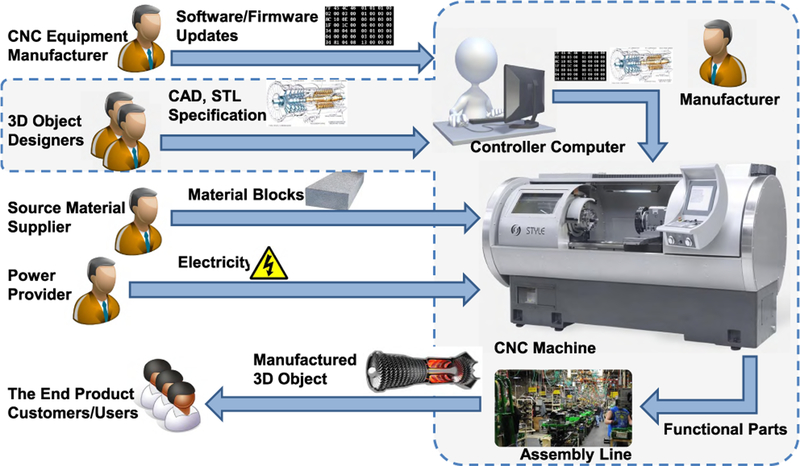
Subtractive manufacturing workflow [27].

Fig. 3 represents a simplified SM workflow for purposes of illustration and comparison to the AM workflow. In this SM workflow, the manufacturer provides a completed product using “in-house” designers. Typically, the manufacturer invests in highly specialized equipment which encourages long-term, high-volume customer relationships. From a cyber perspective, SM software and firmware updates and manufacturing jobs are controlled by computer as in AM; however, the control commands for the SM CNC machines define drilling head movements which remove material as opposed to the AM deposition movements. The material is solid block in SM and auxiliary materials are less important. The SM workflow also includes an assembly line. The assembly line is required to produce parts with obscured internal features since SM is limited to defining external shapes; internal geometry negatively results from the voids created by assembling solid shapes. The components of the SM workflow are typically considered to be co-located and within the manufacturer’s *controlled environment* (indicated by the dotted line area in Fig. 3.

The representative metal AM workflow and simplified SM workflow are typical versions and may not apply to every AM or SM scenario. In the interest of space, we do not attempt to analyze every possible AM or SM workflow configuration. We acknowledge that these workflow representations may not encompass all AM or SM manufacturing scenarios but, rather, present them to facilitate reasonable analysis and discussion.

**FIGURE 4.**
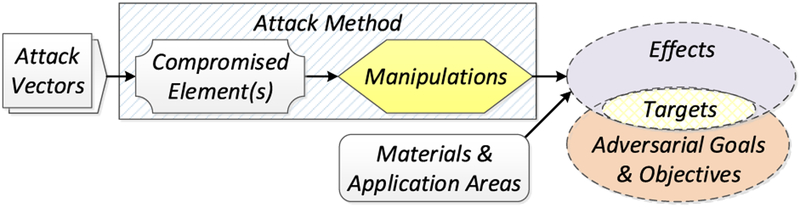
Attack analysis framework [23].

**FIGURE 5.**
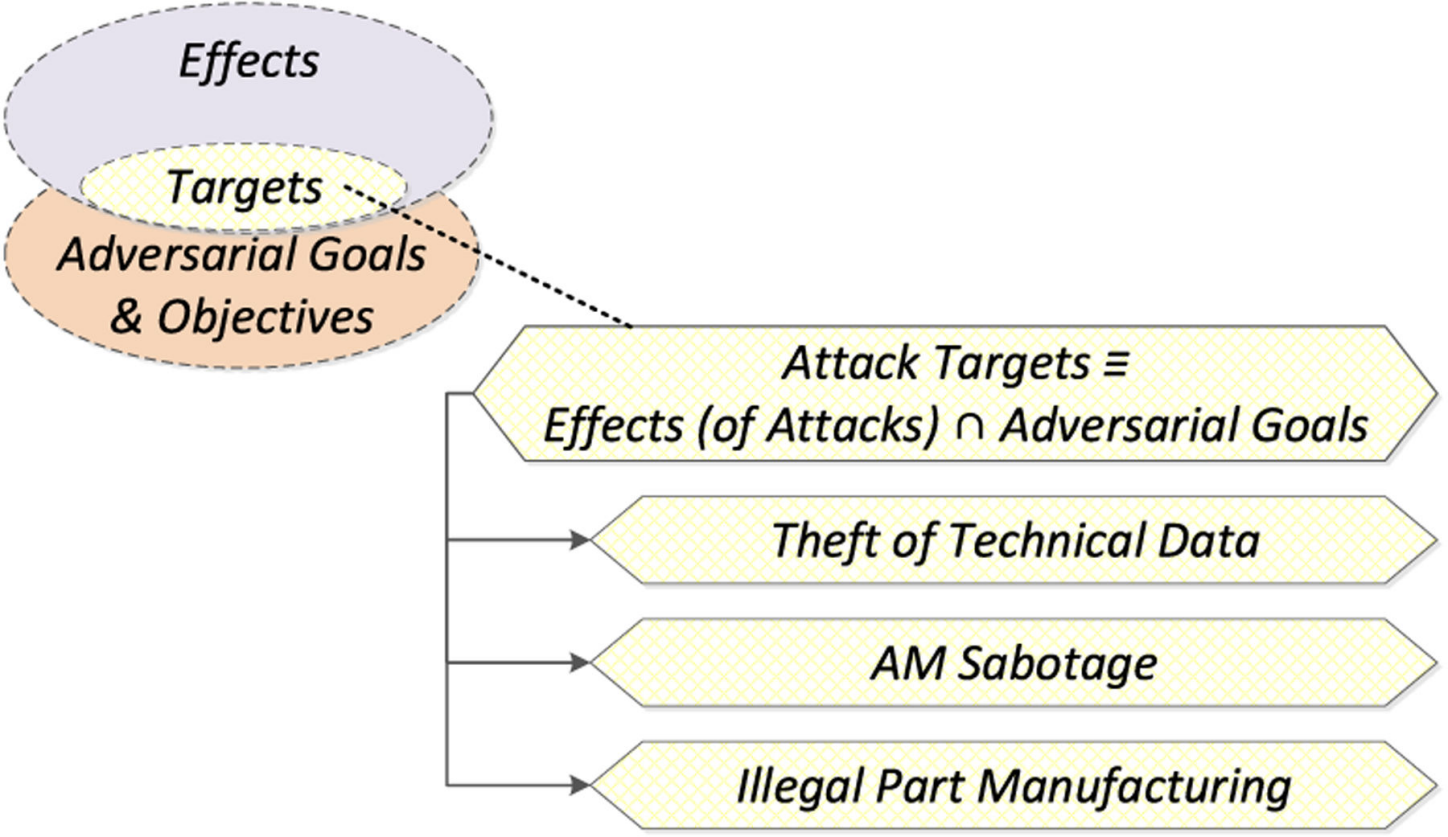
Security threat categories [23].

In our prior work [16], we proposed a framework for the analysis of attacks on or with AM (see Fig. 4). According to this framework, *attack vectors* compromise one or more elements of the AM workflow. The *compromised elements*, their role in the workflow, and the degree to which an adversary can control these elements determine the *manipulations* which can be performed. These manipulations — together with the specific type of AM equipment, source materials, and object application area — in turn determine which *effects* are achievable. Whether an achievable effect is a threat, however, is determined by the adversarial goals which can vary markedly in the AM environment. For example, an effect might prove useful to a counterfeiter but useless to a saboteur. The threat arises therefore from the intersection of the possible attack effects and the specific adversarial goals and objectives. This intersection frames the *attack targets*, which constitute *security threats*. So far, three major security threat categories have been identified for AM: *technical data theft* (or *intellectual property violation*), *AM sabotage*, and *illegal part manufacturing* (see Fig. 5) [23].

Intellectual Property (IP) violation involves gaining access to and unauthorized use of IP. With regards to AM, IP might include 3D object models, the object’s required physical properties (particularly for functional parts), and the AM process specification parameters [28]. Infringement could include producing more than originally authorized or counterfeit products.

Technical data theft can also be a preparatory step for a sabotage attack. AM sabotage attacks can have several goals, which can be accomplished individually or in combination [16]. Examples of different attack goals include part function degradation by failure during operation as demonstrated in the *dr0wned* attack scenario [29], manufacturing equipment damage similar to that of Stuxnet [30], or environmental damage or contamination as discussed in [16].

The last security threat area, illegal part manufacturing, is a growing concern due to the relative ease with which AM equipment can be used to manufacture a wide variety of objects without expensive and time-consuming reconfiguration. Illegal part manufacturing can include *export-controlled* or *nationally or internationally prohibited* items such as firearms or explosive device components [31]–[34]. There is also the potential for AM printers to manufacture instruments of crime, some of which have not yet been conceived nor designed.

### B. ANALYSIS

Our analysis of AM security exposure is organized according to the Attack Analysis Framework [23] (see Fig. 4). The framework components used for this analysis are *attack vectors*, *compromised elements*, *manipulations*, and *targets*. *Attack methods*, which are semantically identical manipulations that can be performed by different compromised elements, and *targets* are analyzed according to the three AM security threat categories of *technical data theft*, *sabotage attacks*, and *illegal part manufacturing* (see Fig. 5).

#### 1) ATTACK VECTORS

While we are not aware of a documented real-world attack on AM systems, exploitation of a variety of attack vectors has been demonstrated or discussed in the AM security research literature. Classic cyber-security attack vectors discussed in the relevant research include spear phishing [35], code injection [36], session hijacking on a Wi-Fi network [37], and cyber supply chain compromise [38]. The cyber supply chain analysis included AM relevant software and technical data.

Additionally, the physical supply chain is another possible attack vector. The AM literature has identified feedstock [38] and the power grid [27], [39] as viable attack vectors.

Generally, all of these attack vectors would be valid for SM as well. Further, some initial distinctions such as machine heterogeneity and Internet connectivity are now applicable to both AM and SM. However, differences between AM and SM security become more apparent when examining the distinct environments in which they operate.

First, the relationships between customers and service providers are different. In SM, due to specialized equipment and operator knowledge, service providers are relatively limited and relationships between customers and providers tend to be stable and long-lasting. In AM, the equipment is more universal, without the same dedicated use restrictions. Additionally, standardization and characterization efforts strive to eliminate machine operator impact. As a result, long term relationships are not necessarily optimal as manufacturers could prefer flexibility to deal with more customers from a potentially unrestricted base and customers can choose among multiple providers responsive to their requirements.

Another difference is the applicable production economics scenario. The specialized machines together with expensive reconfiguration requirements mean that SM is suited for economies of scale scenarios where the customer requires a large volume of identical parts. In contrast, AM is considered an attractive option for low volume, customized, on-demand manufacturing.

Another aspect to consider is the way in which individual machines are used during the manufacturing process. In SM, only external geometry can be modified during the manufacturing process so usually several specialized machines are used to manufacture various components which are then assembled into the final product. In AM, one machine can be used in a variety of ways, replacing the several specialized SM machines. Additionally, in AM very complex internal geometries can be created so the number of components required for the final product can be reduced. As a result, part assembling can be drastically reduced or even eliminated in AM. A frequently cited example is the General Electric (GE) fuel injection nozzle incorporated into the Leading Edge Aviation Propulsion (LEAP) engine. There, AM reduced the number of parts from 20 to 1, eliminating assembly requirements, with a corresponding 25% weight reduction [9].

Lastly, significant differences exist in the final step of the manufacturing environment, the quality control step. In SM, there are several, well-established non-destructive evaluation (NDE) tools, such as Coordinate Measuring Machines (CMM), Structured Light (SL), Eddy Current Testing (ECT), Ultrasonic Testing (UT), and Computed Tomography (CT). CMM and SL measure external geometry, ECT and UT detect internal porosity, and CT detects cracks and internal porosity. Each of these has limited applicability in the AM environment. For example, CMM, SL, and ECT require access to all surfaces of a part [40].

These differences in operating environments have profound implications for AM security as compared to SM security and are summarized in Table 2. The higher cardinality of available AM service providers increases exposure to malicious actors. The short term customer-provider relationships and small volume manufacturing in AM contribute to a temporary, transactional situation in which loss of customers or reputational damage is more situational and less apparent to other customers, increasing the potential for fraudulent behavior. The consolidation of AM technical data throughout the workflow simplifies technical data theft in comparison to SM where the data is divided amongst numerous specialized machines and operators, each only possessing the data necessary to accomplish a specific task. In SM, multiple attacks and targets are needed to obtain comparable information as that which might be retrieved in one targeted AM attack. This also simplifies AM sabotage attacks because a broad variety of manipulations can be staged from the same machine, instead of having to exploit and manipulate multiple, different SM machines. Lastly, the severe limitations of NDE techniques with respect to AM make sabotage attacks impacting internal geometries and part properties harder to detect.

With the impact of environment on attack vectors, it is reasonable conclude that the Return on Investment (ROI) for an adversary is substantially higher in AM than in SM. It is also logical to also conclude that the attractiveness of AM as a target will grow rapidly given the impressive average 26.6% annual growth of the industry over the past 29 years [2] and the expected related decrease in SM as manufacturers transition to AM. Altogether, the exploitability, ROI, and industry growth lead to the reasonable assumption that AM exploit development will be higher than that in SM.

#### 2) COMPROMISED ELEMENTS

The AM security literature has demonstrated the ability to compromise and exploit the workflow elements for different attacks. Past research has examined exploitation of different cyber components of the workflow, such as the controller computer [35], [41], the 3D printer firmware [36], [42], the computer network [37], and digital design files and software components [28], [38]. Physical components have been also considered as a compromisable element which can enable attacks. These include feedstock [38], the power grid [27], [39], and the physical environment [43]–[45].

From these examples, together with the published official incident reports [46] for various industrial control systems (ICS), it is apparent that each and every element in the AM and SM workflows can be compromised. While there is a substantial overlap between AM and SM compromisable workflow elements, further examination indicates there are also significant differences.

Some compromised elements can be categorized as cyber domain specific such as design files and process requirements. While AM facilitates dynamic on-demand customer-provider relationships, it correspondingly creates greater exposure avenues for the digital design files and requirements specifications. The files can be benign initially and then compromised while part of the digital chain or they can originate from a malicious actor. In either case, in addition to being compromised elements, they can function as attack vectors to compromise the service provider environment. Additional cyber components include the process modeling software used to simulate and correct geometry distortion resulting from AM-induced effects such as warpage as well as the computer-aided design (CAD) software and toolpath generators. Compromise of this software can have profound security implications, both enabling a broad range of sabotage attacks and also affording unfettered access to technical data.

Other compromisable elements in the AM workflow can be categorized as physical domain specific such as the commodities used during the manufacturing process. These include the source material, called feedstock, and auxiliary materials supporting the manufacturing process such as inert gas and power. While power is not unique to AM, power grid manipulation impacts the AM manufacturing process and final part quality in a far different and more significant manner than SM. For example, in AM power fluctuations can change the melting properties, detrimentally impacting fusion and resulting in part loss as opposed to SM where power fluctuations can mean simply a temporary work stoppage.

Compromisable cyber-physical elements in the AM workflow include actuators and the associated signals and commands. It also includes sensors and the transmitted data. In both cases, the physical component can be modified or damaged or the data produced and exchanged by the physical component can be compromised, altering system performance. A sensor compromise example for AM would be infrared (IR) thermography for quality control as demonstrated by Slaughter *et al*. [47]. With IR thermography, false sensor data can be generated by damaging the sensor or altering the data it generates. In the Slaughter *et al*. [47] scenario, faulty data allows a bad quality part to satisfy quality control requirements.

While such cyber-physical compromises can be relevant for SM too, some compromisable elements in the AM workflow have no or significantly less relevance for SM cyber security. These include feedstock characterization, powder recycling, and post-processing heat treatment. Table 3 summarizes the compromisable AM workflow elements.

There are also elements in SM workflow that are either not present in AM workflow or have very little relevance to its security. Most notable is the assembly step. In SM, the restriction to shaping external geometries necessitates multiple part assembly to create parts with internal geometries. AM drastically reduces the extent to which assembly is needed, sometimes eliminating it entirely, due to its ability to create internal geometries during the fusion process. Another notable aspect is toolpath generation. In multi-axis SM machines, the toolpath generation software has to account for possible collisions between the various moving parts of the machine and the manufactured part. In AM the avoidance of such situations is considered trivial.

#### 3) ATTACK METHODS AND EFFECTS

From each individual compromised element, various manipulations are possible. However, for the sake of concise discussion, it is possible to group semantically identical manipulations. An example of such a grouping would be part geometry modification, which can be accomplished through STL file manipulations, toolpath command alterations, compromise of the digital representation in firmware or memory during processing, or even modification of individual actuator signals. Together, the semantically identical manipulations that can be exercised by different compromised elements are referred to as *attack methods*, depicted in the Analysis of Attack Framework [23], discussed in subsection III-B and depicted in Fig. 4. *Attack methods* and their *achievable effects* are distinct for the different threat categories. Therefore, we organize the discussion below by the threat categories of *technical data theft*, *sabotage attacks*, and *illegal part manufacturing* (see Fig. 5).

##### a: TECHNICAL DATA THEFT

In the AM context, unauthorized use of relevant part production data is often referred to as intellectual property (IP) theft. However, not all technical data parameters essential to produce a quality product are protectable under current United States (U.S.) intellectual property law [48]. For our discussion, technical data theft refers to legally protectable IP as well as the information necessary to produce the part such as process parameters.

The technical data theft attacks examined in AM security research literature thus far can be categorized as solely cyber and cyber-physical. Demonstrated cyber attacks have included a spear phishing attack used to exfiltrate an STL file ^6^ [29], [35] and network session hijacking used to retrieve the last printed model [37].

Cyber-physical attacks demonstrated in the literature have used physical emanations, also referred to as side-channels, to reverse engineer the AM part and related technical data. Demonstrated cyber-physical attacks have included single side-channel stepper motor acoustic emanations which were able to reconstruct a part with 85.72% accuracy [43] and combined acoustic and electro-magnetic emanation side-channels [44], [45].

**FIGURE 6.**
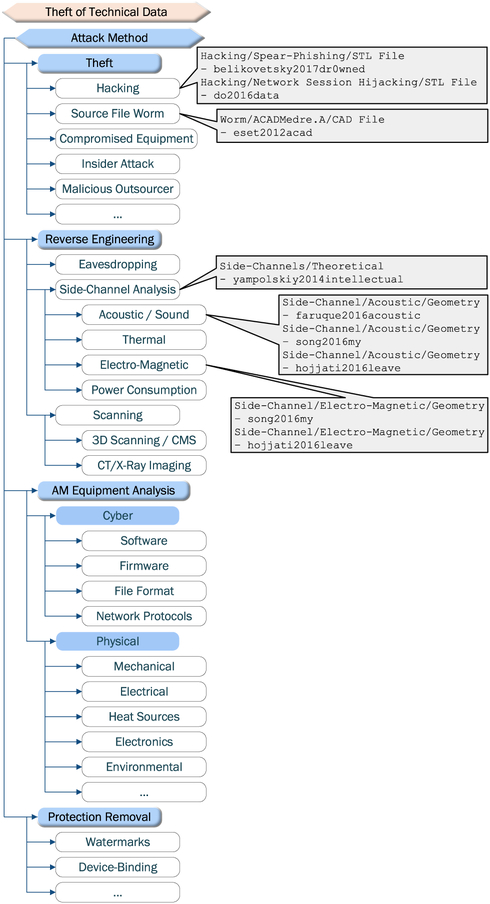
Technical data theft methods (derived from [23]).

The manipulations shown in the research literature constitute only a sub-set of attack methods identified and categorized by Yampolskiy *et al.* [23] (see Fig. 6). It is arguable that some of the cyber and cyber-physical attack methods listed in the taxonomy for technical data theft can be applied to SM as well. We summarize similarities and differences in Table 4.

There are, however, differences in the difficulty level required to achieve desirable results in AM as opposed to SM. With methods like scanning using structured light or high precision CMM machines, technical data theft can be easier for SM than it is for AM. This is due to the fact that SM only defines exterior geometry, which is easily discernible by these methods, while AM defines both external and internal geometry as well as the part microstructure. At this time, precise scanning of internal cavities still poses a significant challenge, even with CT, rendering these methods ineffective for reconstructing AM internal geometries as well as the microstructure. Additionally, AM includes technical data required to produce quality-specified material during the manufacturing process [28]. This integral information, required to produce a specific AM part, can be obtained through classic cyber means as part of a technical data theft attack. In SM, however, the material is not produced as an integral component of the part manufacturing; it is produced prior to beginning part manufacturing and, as such, the material-specific quality information is segregated in the workflow, residing with the material supplier, as opposed to the continuous AM digital thread.

##### b: SABOTAGE ATTACKS

While sabotage attacks have yet to be documented in real world scenarios, those shown in the research literature can be grouped into two categories: *direct* and *state estimation* attacks. Table 5 contains a summary listing of these attacks together with the corresponding reference citations.

Several direct attacks manipulate specifications by changing part representation such as modifying the external shape [36], [41], substituting a different object [42], introducing internal cavities [29], [35], [41], or incorporating contaminant material [49].

Other direct attacks presented in the research literature manipulate the manufacturing process in order to damage the part. One such attack included changing the build direction which leveraged anisotropic^7^ properties to detrimentally impact the part mechanical properties without modifying part geometry [38], [49]. Other proposed or demonstrated process parameter attacks include scanning strategy,^8^ heat source speed and targeting, and power intensity [38].

Workflow process attacks include communication timing and power supply manipulations [27], [39] and supply chain attacks [38]. Supply chain attacks include feedstock manipulations such as changing the powder chemical composition or geometric characteristics. While feedstock manipulations can have a direct impact on quality, they are indiscriminate and cannot be used to target a specific part [16].

The state estimation attack demonstrated in the research literature involved manipulating infrared (IR) thermography sensor data (see Table 5). In an AM system using an IR feedback control loop, manipulating the IR sensor data can be used to influence the manufacturing process parameters and ultimately part quality [47].

**FIGURE 7.**
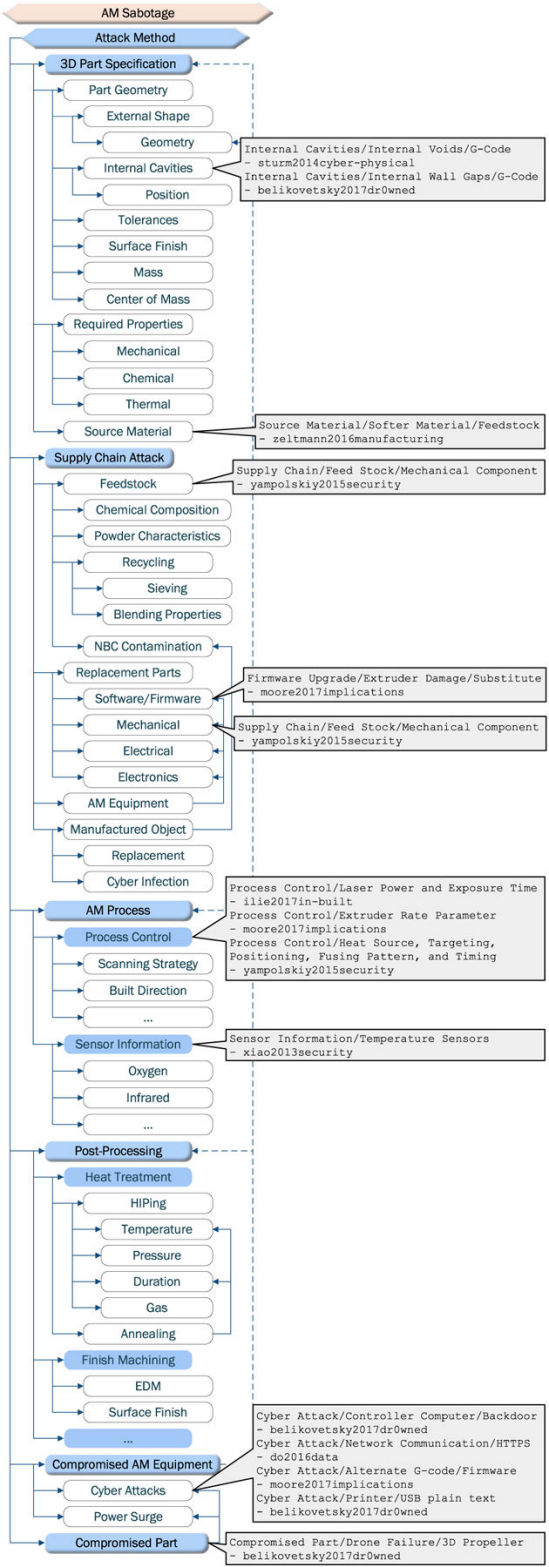
AM sabotage methods (derived from [23]).

Of all the proposed or demonstrated AM manipulations, only changes to external shape can be replicated by SM machines. All the remaining manipulations are unique to AM (see Table 5). It is noteworthy that the manipulations shown in the research literature constitute only a fraction of attack methods identified and categorized in a taxonomical form by Yampolskiy *et al.* [23] (see Fig. 7).

##### c: ILLEGAL PART MANUFACTURING

Illegal part manufacturing does not fit precisely into the analysis framework. Illegal part manufacturing can be defined in AM as manufacturing a part without authorization or manufacturing a prohibited item. Without authorization can include manufacturing more items than originally licensed as well as without any authorization at all. Currently, there are no safeguards to prevent such manufacturing and thus no attacks to thwart them. When protections are implemented, then an attacker will need to either remove or bypass them and that situation would then be analyzable using the framework. Until that point, manufacturing without or exceeding authorization invokes the same considerations as technical data theft — the data is being used in an unauthorized manner in both instances and both instances require data access, only how the data was originally obtained varies.

#### 4) ATTACK TARGETS

While an *attack method* will have an *effect*, it may not be the desired effect depending on the *adversarial goals and objectives*. The intersection between the adversarial goals and objectives and the achievable effects define the *attack targets* (see Fig. 4). As with *attack methods*, *attack targets* are distinct for the different threat categories.

##### a: TECHNICAL DATA THEFT

The technical data theft attacks demonstrated in the AM security literature have been focused on the part design file. The spear phishing and network session hijacking attacks focused on STL file exfiltration [29], [35], [37]. The STL file is currently the most widely used AM file format; the format is limited to part geometry specification. The side-channel attacks reconstructed (with varying degrees of error) part geometry only [43]–[45].

Obviously, part geometry specification is of interest in both AM and SM cyber-security contexts. However, in the AM context, valuable technical data is not limited to geometry alone. In the AM context, part material characteristics are defined along with the part geometry. While the design is essential to achieve the geometry, the manufacturing process parameters are essential to achieve the required part properties. Thus in the AM context, technical data also includes the required part properties specifications and manufacturing process parameters specifications [28]. These aspects are widely irrelevant for SM, because during the SM manufacturing process only external geometry can be specified; the material characteristics are determined separate from the part manufacturing process.

**FIGURE 8.**
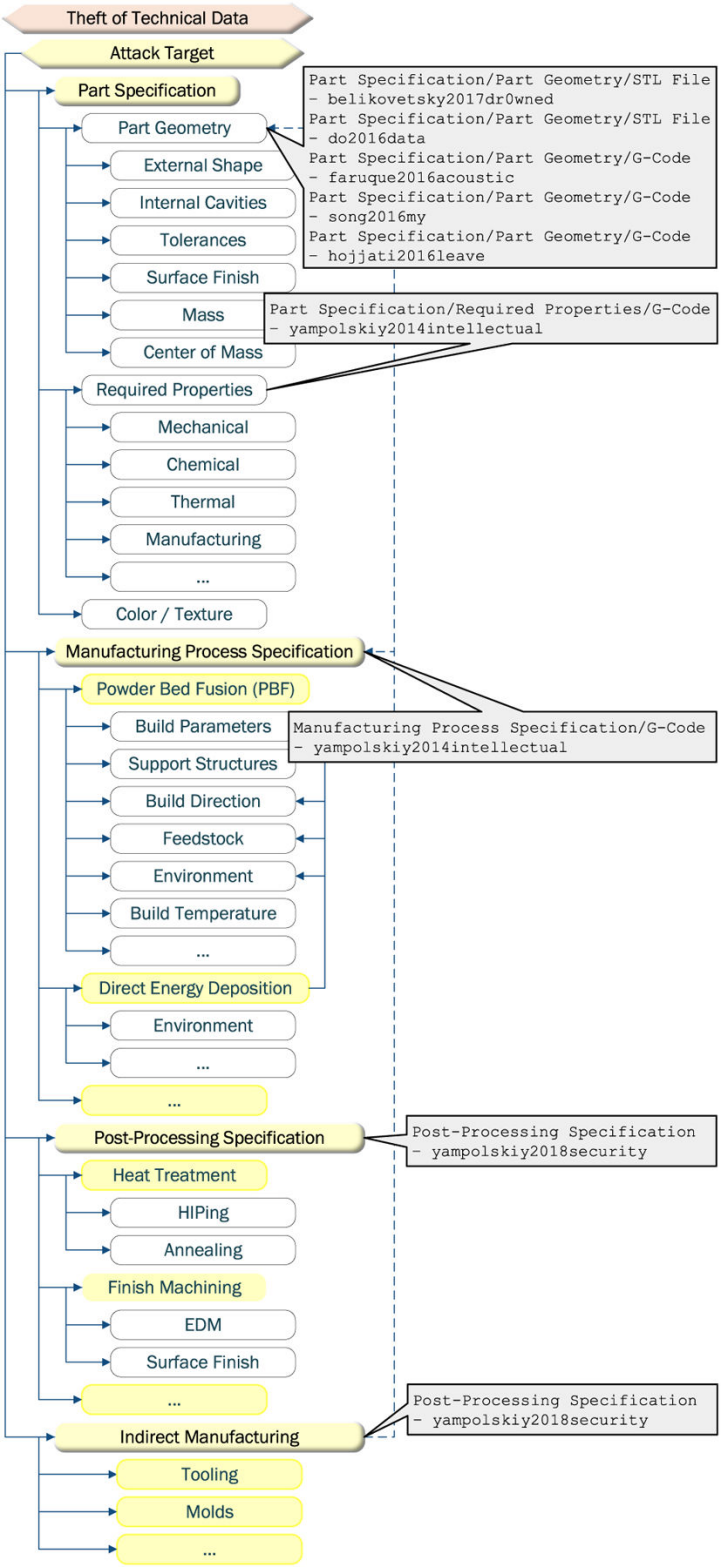
Technical data theft targets (derived from [23]).

Other possible technical data theft attack targets might be *post-processing specification* and *indirect manufacturing* such as tooling (see Fig. 8). While both can be used with SM as well, their role is less dominant. For example, heat treatment post-processing operations such as *hot isostatic pressing* (HIPing) and *annealing* are often needed to release internal residual stress in metallic AM parts. However, they are rarely needed in SM.

We summarize the attack target similarities and differences in Table 6.

##### b: SABOTAGE ATTACKS

Sabotage attacks can target the manufactured part, the AM equipment, or the environment of either the part or equipment [16], [23]. For the manufactured part, the attacks demonstrated in the research literature have targeted degrading part tensile strength in order to induce breakage under a lower mechanical load [41], [49] and increasing material fatigue development in order to induce breakage prematurely after a period of normal operations [29], [35].

AM equipment attacks have been discussed in literature where the researchers [16] theorized that it is possible to increase equipment wear and eventually achieve physical damage in a manner similar to that of the Stuxnet attack [30]. A part environment attack was demonstrated in the *dr0wned* attack where a quadcopter drone was destroyed by sabotaging one of its 3D-printed propellers [29], [35]. An equipment environment attack was theorized as a potential danger based on the materials used in metal AM [16]. Metal AM powders are very fine, considered generally hazardous, and can be combustible depending on the alloys [16], [50]. The damage potential was accidentally demonstrated on November 5, 2013 at a Powderpart 3D printing plant in Woburn, Massachusetts where several safety standards violations resulted in a combustive dust explosion and small fire [16], [51].

**FIGURE 9.**
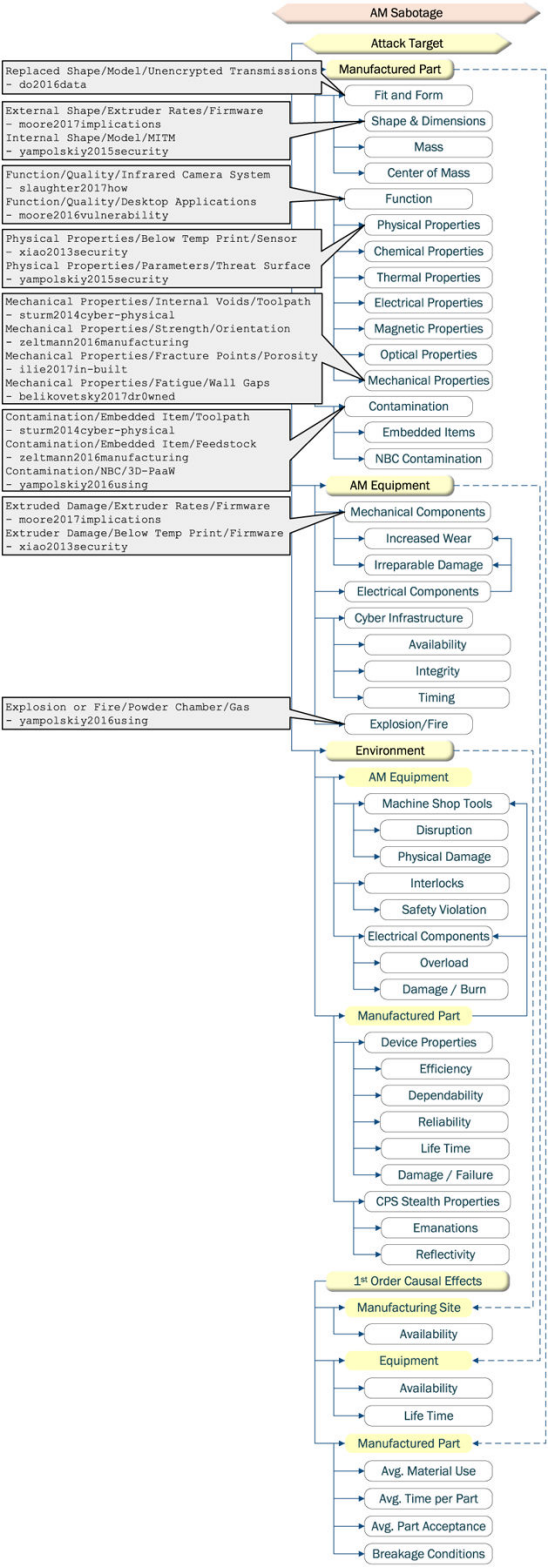
AM sabotage targets (derived from [23]).

Looking at the categories only, one could say that all but the impact on the AM equipment environment are achievable in SM as well (see Fig. 9). However, under closer inspection, attack targets achievable in SM are only a sub-set of attack targets achievable in AM (see Table 7 for the summary). It is possible to degrade tensile strength in SM. However, the required defects will be changes of external geometry and, as such, easily detectable by quality control measures. In AM, the defects can be introduced through internal geometry changes as well external alterations or by altering material properties through modified manufacturing process parameters. Currently existing non-destructive testing (NDT) measures are severely limited in detecting internal or material-related defects [40].

##### c: ILLEGAL PART MANUFACTURING

As mentioned previously in paragraph III-B.3.c, illegal part manufacturing does not fit precisely within the current attack and target analysis framework. With the rapid and wide-spread adoption of AM, there are growing concerns about use of this technology for illegal part manufacturing. The concerns include the manufacturing of undetectable guns and nuclear components [52]–[54] and the use of AM to circumvent export control regulations and nuclear non-proliferation treaties [52]–[54]. While illegal part manufacturing has been achievable with SM, some researchers argue that AM will significantly simplify this process [52]–[54].

Several researchers have also investigated and proposed measures which could be used to prevent illegal part manufacturing. Such measures include using an image database system to recognize authorized part manufacturing and halt unauthorized print jobs [55], using surface texture variations to trace objects to a specific printer [56], and design file watermarking [57], [58]. Once preventative measures are implemented, then the attack analysis framework can be extended to identify and address the illegal part manufacturing targets.

Our analysis of methods and attacks according to the three threat categories shows that overall, while there is an overlap between AM and SM security, numerous aspects are unique with some differences arising from the technology itself and some from the manufacturing environments.

## IV. PERSPECTIVE II: EVALUATION

In this section, we outline the background necessary to understand system and risk assessment. Then we provide a system assessment analysis indicating where steps and procedures are unique to AM or also applicable to SM.

### A. BACKGROUND

**FIGURE 10.**
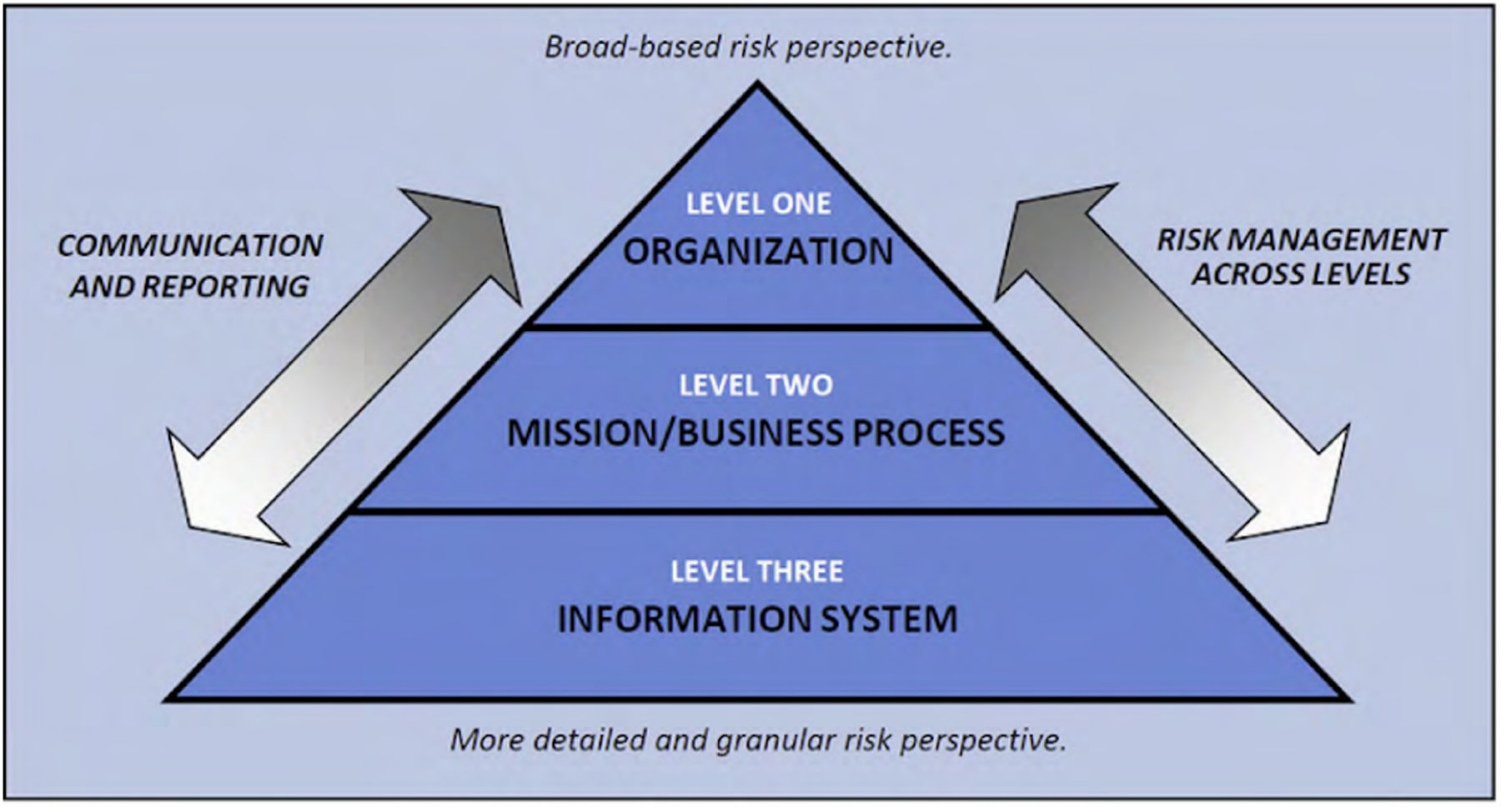
Organization-wide risk management approach [21].

There are various methods which can be used to assess a system and the robustness of its security posture. The National Institute of Standards and Technology (NIST) has developed the Risk Management Framework (RMF) [21] for managing risk at the organizational and system level using a well-ordered process approach. The framework is based on industry best practices; it is not designed to address and prevent all possible attacks but rather to help organizations develop systems and processes which can address the known, worst-case scenarios and to recover quickly and resiliently from all others such as zero-day attacks. Fig. 10 details the RMF organization-based approach which places system assessment as the foundational level of risk management. Systems may include not only desktop computers, laptops, and servers, but also networked devices like AM machines and the other operational technology (OT) depicted in the representational AM workflow depicted in Fig. 2. Furthermore, a system is not limited to a single device. For example, the AM machine and controller computer in Fig. 2 could be logically grouped together as a system.

**FIGURE 11.**
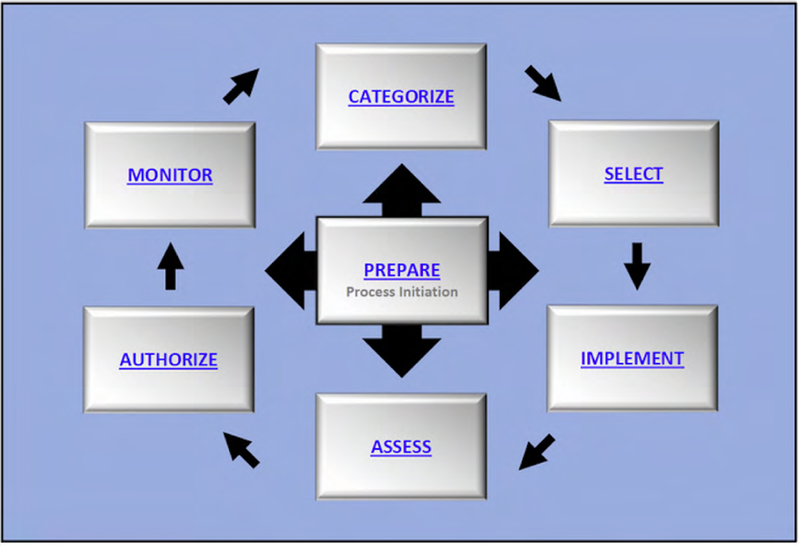
RMF steps [21].

**FIGURE 12.**
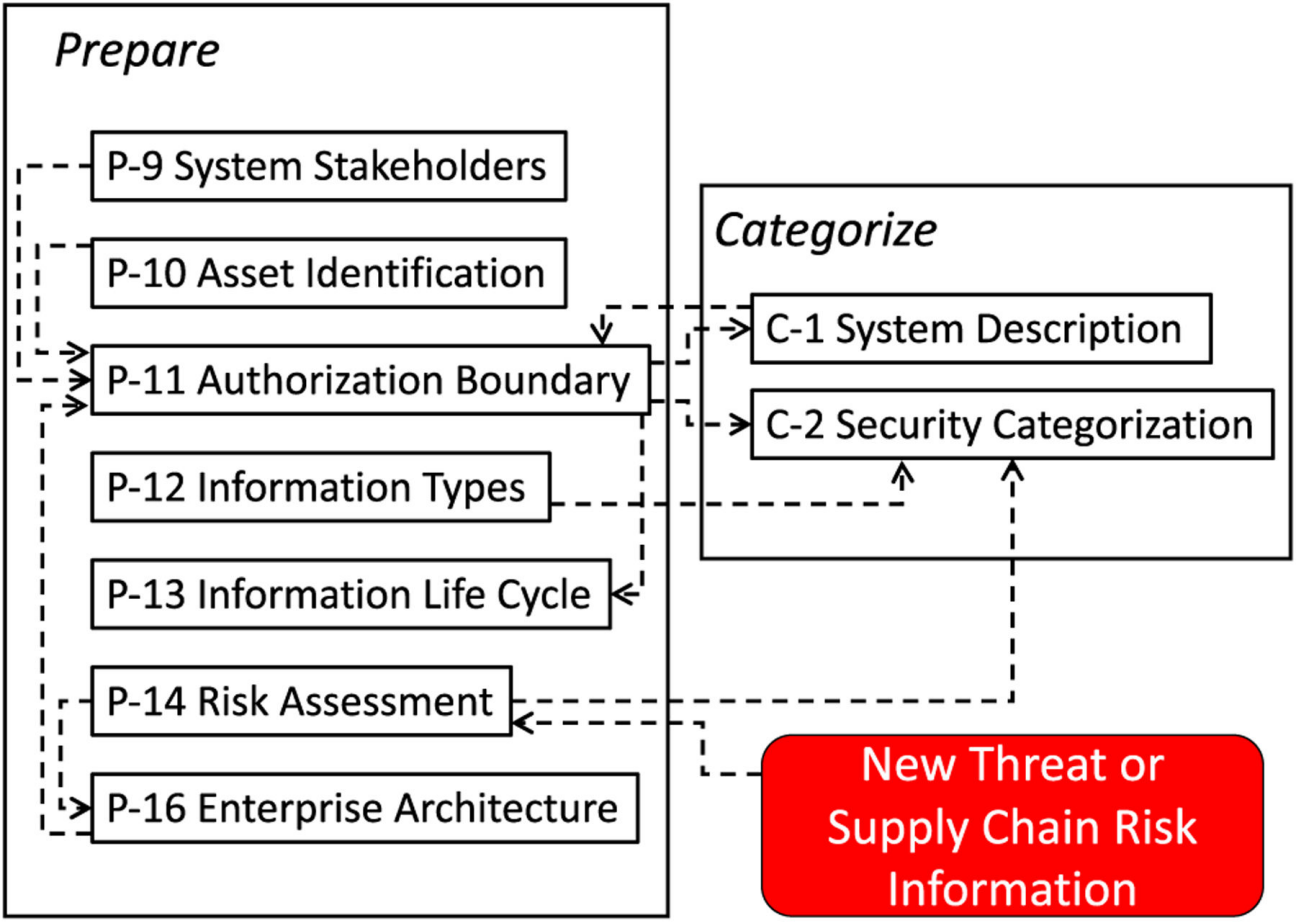
*Task P-11* inter-dependencies within *Prepare* and with *Categorize* steps.

The RMF process is organized according to steps and tasks. The steps at the system level are *Prepare*, *Categorize*, *Select*, *Implement*, *Assess*, *Authorize*, and *Monitor* (see Fig. 11). Each RMF step consists of multiple tasks and each task has a set of potential inputs, many of which are outputs from other tasks. As an example, Fig. 12 depicts interdependencies within and between the *Prepare* and *Categorize* steps and tasks.

While the framework is designed to apply to all systems, the actual task outcomes are unique, depending on specific business requirements, risk tolerance, budgetary constraints, enterprise architecture, and other factors. Some inputs and outputs can be used to develop Cybersecurity Framework (CSF) Profiles. The “Framework Profile” can then be employed to represent the current or desired state of cyber-security activities. One example of a profile is the Cybersecurity Framework Manufacturing Profile [59], which could potentially be applicable to AM^9^ systems.

The RMF encourages streamlining the process through organization-wide common security mechanisms and aggregation of individual components into a “system.” Component aggregation into a system is done using *authorization boundaries* established by *authorizing officials* in *Prepare Task P-11* [21]. The RMF defines an authorization boundary as the establishment of “the scope of protection for an information system (i.e., what the organization agrees to protect under its direct management or within the scope of its responsibilities)” [21, p. 17].

The system authorization boundary determination then in turn provides input to the *Categorize* step. *Categorize Task C-1* is the system description. *Task C-2* is the system security categorization where each system information type is assessed according to its potential impact on confidentiality, integrity, and availability. Broadly, the *Categorize* step is used to determine the level of “the adverse impact to organizational operations and assets, individuals, other organizations, and the Nation with respect to the loss of confidentiality, integrity, and availability of organizational systems and the information processed, stored, and transmitted by those systems” [21, p. 46]. This impact level determination defines the appropriate security control baseline which is an input to the *Select* step.

Once the *Categorize* step is completed, the process moves on to the *Select* step. During this step, the system owner selects security controls appropriate for the system and its operating environment according to the control baseline. There are over 800 security controls and enhancements grouped into 18 families [62], [63] in the security control catalog for the system owner to evaluate for possible selection.

The *Prepare* and *Categorize* steps form the basis for the security decisions made in the later steps, such as selecting which security controls and enhancements are applicable and how they should be tailored. They are also where the differences between AM and SM security under RMF are distinct and most impactful. Once the AM “system” is established under those two steps, the control selection process will be similar for both manufacturing processes. Implementation related concerns are discussed in Section V.

The pertinent *Prepare* tasks as highlighted in Fig. 12 are P-9: System Stakeholders, P-10: Asset Identification, P-11: Authorization Boundary, P-12: Information Types, P-13: Information Life Cycle, P-14: Risk Assessment - System, and P-16: Enterprise Architecture. P-14 is triggered by new threat information as was previously discussed in Section III. The pertinent *Categorize* tasks are C-1: System Description and C-2: Security Categorization. The tasks and their outcomes are summarized in Table 8 and Table 9. The tables provide the task identifier and name, expected out-come, and CSF equivalents (if any). The interdependencies within and between the *Prepare* and *Categorize* tasks are depicted in Fig. 12.

### B. ANALYSIS

**FIGURE 13.**
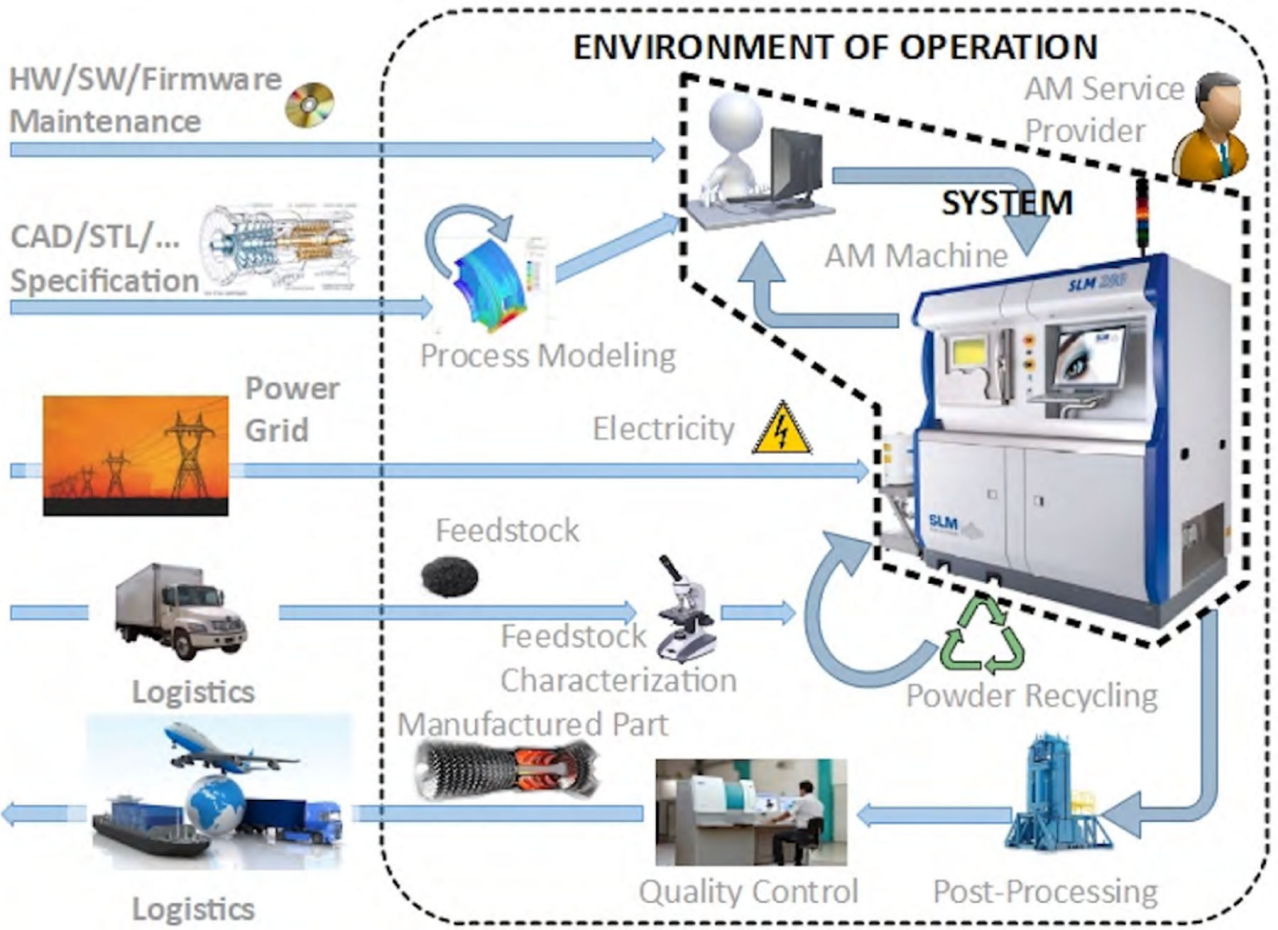
AM machine and controller computer as a system (derived from [27]).

For our analysis we consider a scenario where an AM service provider with workflow shown in Fig. 2 establishes the authorization boundary shown in Fig. 13. The authorization boundary is delimited by the coarsely-dashed line. This authorization boundary defines what we shall refer to as “System AM,” a system whose elements are the AM machine and its controller computer.^10^ The system’s larger environment of operation,^11^ delimited by the finely-dashed line, includes systems outside the authorization boundary that are within the AM service provider’s organization. Some of these systems, such as the feedstock characterization and process modeling systems, are enabling systems that provide support to the authorization boundary-delimited system. Other systems outside the environment of operation, such as the feedstock supply chain and power grid, also provide support.

Feedstock supply chain disruption poses a major threat to an AM service provider. Multiple attack vectors can aim at the AM feedstock supply chain, as discussed in Section III. Notably, source material vulnerability is more pronounced in AM than in SM. In SM, the source material is a solid block, making it hard to attack the material beyond its surface. In AM, however, a feedstock attack can employ a variety of methods, as shown in Fig. 7. Even if an AM service provider performs in-house feedstock characterization, state-of-the-art characterization nowadays is cyber-dependent. For example, computer vision algorithms are now used [64], and these algorithms may be implemented using software applications and libraries which themselves are attack targets that can be compromised to provide incorrect results or behave otherwise maliciously.

In AM, a possible feedstock compromise will trigger a System AM risk assessment. To evaluate the AM-specific impact, we consider two threat cases:

1. It has been reported that the feedstock supplier has been the victim of a cyber-attack.
2. A vulnerability has been reported for the image processing software used by the organization to characterize the supplier’s feedstock.

For case 1, the risk assessments offer the AM service provider four alternatives. First, do nothing and rely on the in-house characterization operation to detect any feedstock issues. Second, change feedstock suppliers. This may not be a viable alternative due to limited vendors and does not address the status of the current stock. Third, establish control over the feedstock supplier’s cyber-security measures. This can include inspection and monitoring of the feedstock supplier together with contractual security compliance requirements. The additional measures and requirements will increase security costs for both parties without guaranteeing against another incident. Fourth, bring the feedstock supply chain in-house either through establishing a native capability or expanding through acquisition of a supplier. Such an option could be cost-prohibitive.

For case 2, the risk assessments offers the AM service provider three alternatives. First, install a patch that fixes the image processing software vulnerability, assuming one is available. Second, replace the image processing software with a non-vulnerable alternative. This alternative raises security-related operational expenses and relies on the fallacy that a currently non-vulnerable alternative will remain non-vulnerable. Third, abandon in-house feedstock characterization, and instead rely on the feedstock supplier’s characterization to detect anomalies. Under this scenario, the service provider delegates control which may make it more susceptible to a case 1 incident.

Our feedstock compromise scenario shows the difficulty the AM service provider has in securing its system under traditional security concepts such as authorization boundaries. For the service provider to adequately address security issues, it needs control over workflow components which may or may not be within operational control. To add further complexity, the area of operational control and security risk can change for the service provider if it fulfills more than one role in the manufacturing-supply chain or if its role changes depending on job. Each change triggers the need for another risk assessment with each assessment consuming additional organizational resources of time, money, and effort.

While SM is subject to cyber attacks, the SM environment is stable without the complexity of shifting system boundaries or job- and role-dependent expanding and contracting attack surfaces. Additionally, AM has the digital thread exposure necessitating that the workflow participants become concerned with and take responsibility for the security of up- and down-stream business partners. As a result, security concerns and costs become higher with a different cost-benefit weighting in AM security.

## V. PERSPECTIVE III: IMPLEMENTATION DECISION

Subsequent to each system assessment cycle, decisions must be made as to which security measures to implement. Due to resource constraints and time-to-market pressures, it will usually not be feasible to implement all security recommendations at once or completely. Additionally, in the AM environment, security measures can impact functionality or interfere with hard real-time requirements that are essential for the proper outcome of the manufacturing process. The recommendations are thus evaluated and prioritized which necessarily includes liability exposure and cost-benefit analyses.

### A. BACKGROUND

When considering which security measures to implement, organizations must consider the cost and the benefit given restricted resources. The cost can be immediate as in expenditures for anti-virus protection or more removed as in future litigation expenses. The cost can also be indirect as in an impact on process efficiency from equipment downtime. The benefits can be immediate such as preventing IP theft or part sabotage or more removed such as preventing loss of reputation and liability exposure. The benefits can also be indirect such as obtaining contracts based on documented security posture maturity.

The costs and benefits for each organization will depend on the perceived AM security threat category and potential consequences. For example, in a technical data theft scenario, not all data is equally valuable to an organization. Part dimensions can be extremely valuable for a company with a limited, unique product line but relatively less valuable for a non-unique part produced by multiple vendors. However, the value in the latter situation may increase if the dimension data can be used to launch a sabotage attack with or against the final finished product. The potential consequences can vary from catastrophic failure of a safety-critical part resulting in loss of life to decreased market share or lost business relationships.

The liability exposure can vary depending on the injured party. When the damage is between participants in the manufacturing process chain, indemnity is often addressed contractually as a negotiated cost between the parties. An example could be liability of the AM machine service provider to the part designer for files stolen during an internal network compromise. Liability to a party outside the manufacturing process chain could occur when an end user or bystander is personally injured in a sabotage attack [65]. Various theories of liability include product liability, warranty, and negligence [65]. Criminal liability for business actions is possible. However, civil liability is usually more punitive and the focus when dealing with companies. An example is the Deepwater Horizon incident where the relatively small criminal fine of $1.256 billion can be compared to the larger civil fines of $18.7 billion [65]–[67].

**FIGURE 14.**
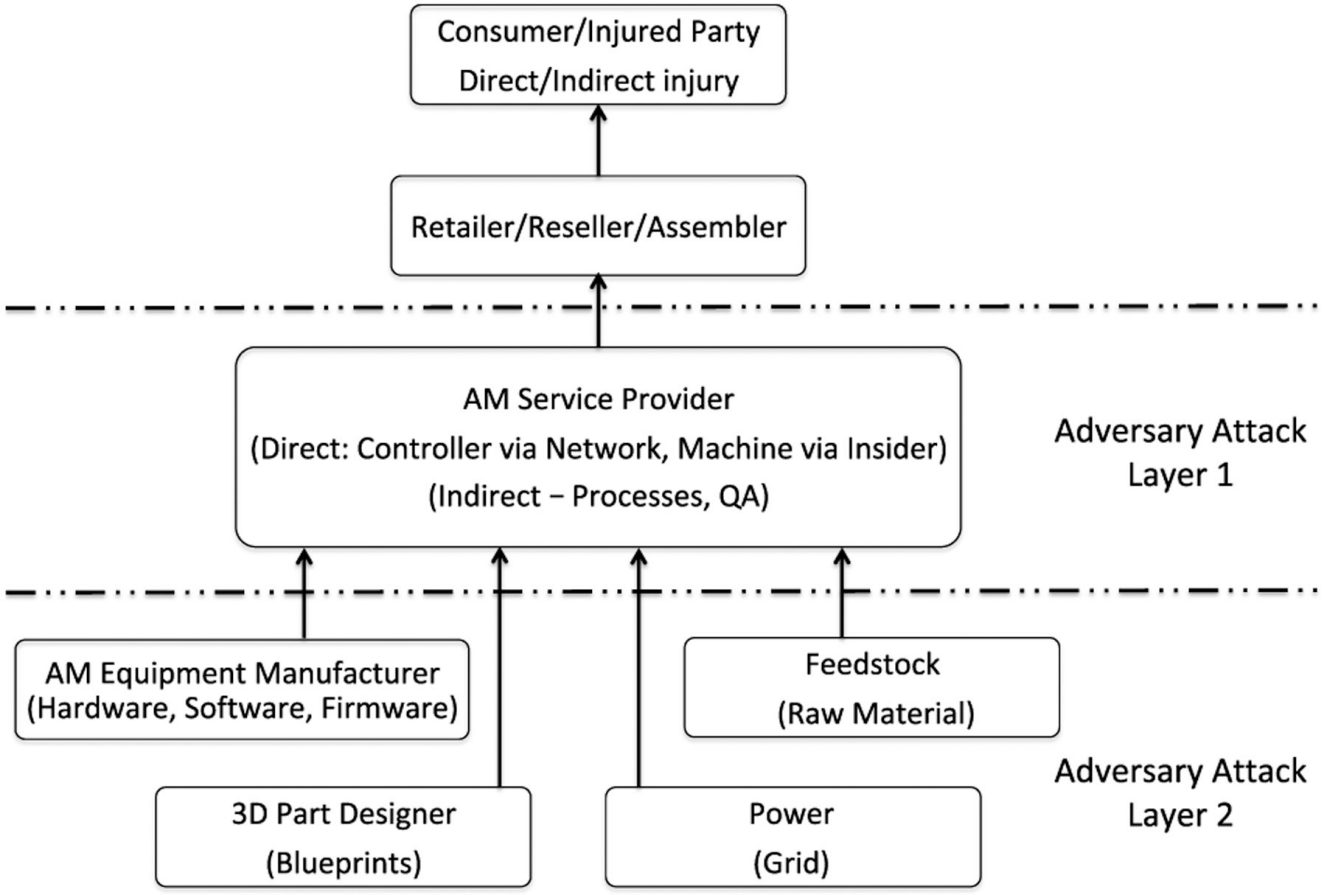
Sabotage attack liability analysis framework [65].

The unique features of the AM workflow environment and the applicability of personal injury liability have been discussed in the research literature. Potential defendants have been identified as the part designer [68], the CAD file generator [69], the part printer [69], the printer manufacturer [68]–[70], and online and print service providers [70], [71]. AM has alternately been categorized and analyzed as an authorized dealer distribution chain [72] and a supply chain [73] to identify the potential parties and scope of liability. Fig. 14 presents an analysis framework which we proposed and used in an earlier work to analyze sabotage attack liability exposure [65]. The framework separates the AM workflow participants into adversary attack layers. The attack layers are separated between manufacturers and suppliers and characterized by targeting ability and scope and by distance from an injured end user. Potential theories of liability have been identified as strict products liability [68], [69], [72], [74], breach of warranty [72], [74], and negligence [72], [74].

**FIGURE 15.**
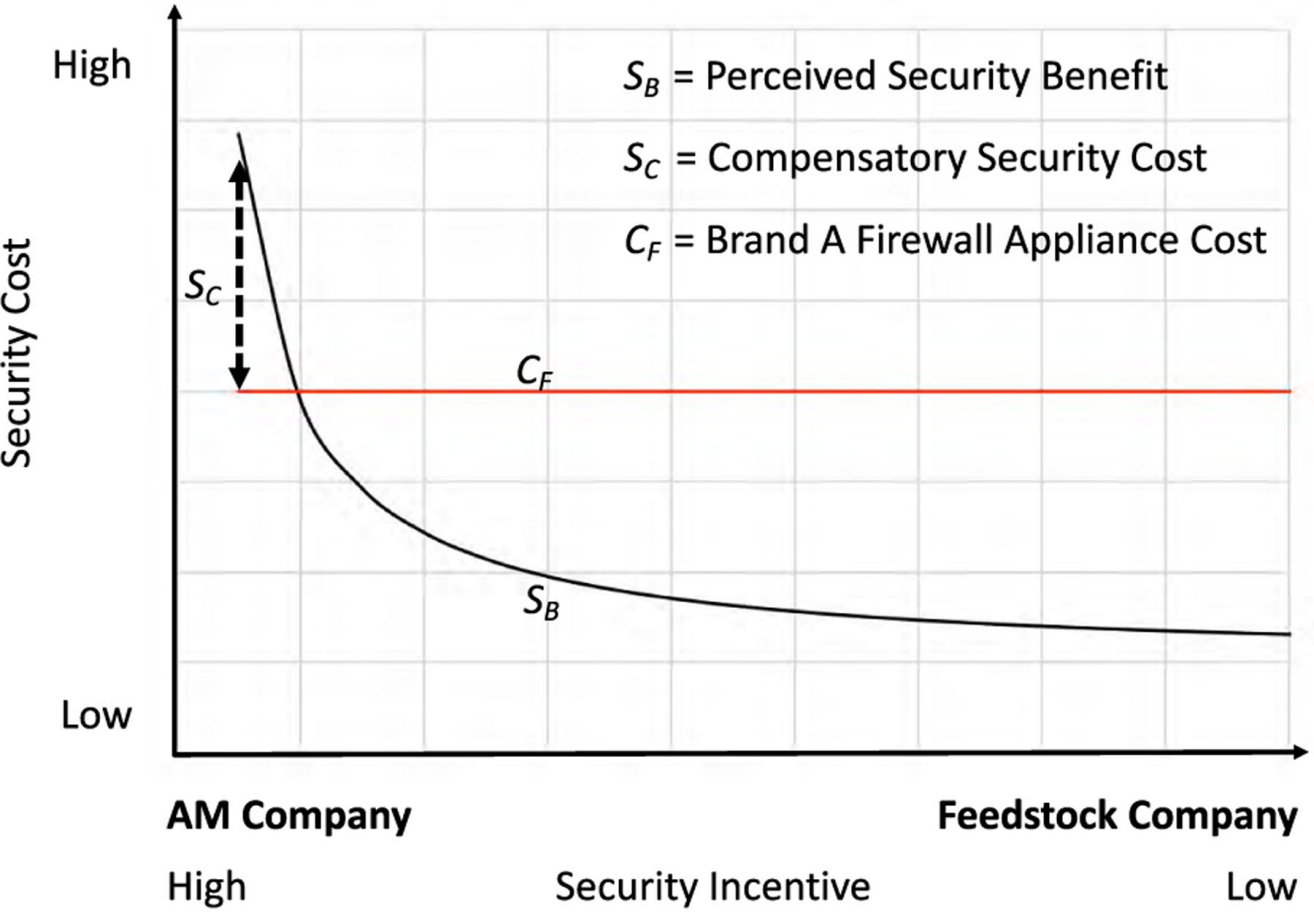
Security cost-benefit weighting.

Based on the potential liability, there is a different cost-benefit weighting for different organizations. Those facing less liability exposure experience less benefit for the same cost and arguably have less incentive to implement security while those facing high exposure must assume a higher cost to compensate. For example, the cost of Brand A Firewall Appliance is the same regardless of liability potential. AM Company recognizes the potential liability from a sabotaged part resulting from an unsecured network so for it the benefit is high. Feedstock Supply Company would be further removed from liability for the same incident, therefore its benefit is lower and it has less incentive to invest in the same security. However, Feedstock Supply Company could be an access conduit to AM Company.^12^ This results in AM Company experiencing a higher security cost as it must provide its own security against the malicious actors as well as additional measures to account for uncertainty in business partner security. The additional measures could include vendor-site inspections and verifications, additional insurance, or additional cyber defense measures. See Fig. 15 for a graphical representation of the possible cost-benefit weighting.

### B. ANALYSIS

The AM workflow depicted in Fig. 2 represents a generalized workflow. Due to the various, flexible ways in which AM workflow components can be organized, from separate entities to integrated service providers and different combinations in between, it is beneficial for purposes of discussion to organize the various participants in a generalized grouping. One possible organizational grouping was presented in the analysis framework depicted in Fig. 14 which was used in our prior work on sabotage attack liability exposure [65].

In the attack analysis framework, the AM workflow participants are separated into adversary attack levels. Attack Layer 1 contains the manufacturing level components of AM service provider, including any assembly. Attack Layer 2 contains the commodities level components such as firmware and software, blueprints, raw material, and power. In our security analysis presented in this section, we exclude internal malicious bad actors and assume the measures address the three AM threat areas — technical data theft, sabotage attack, and illegal part manufacturing.

#### 1) ATTACK LAYER 1

Attack Layer 1 consists of those workflow components recognized as manufacturing. This level includes the AM equipment and the controller computer as well as any assembly or post processing. When considering which security measures to implement, the AM service provider evaluates the attack targets.

##### a: TECHNICAL DATA THEFT

As the AM service provider evaluates and prioritizes the security measures to implement, it must consider the value of the information to be protected. In the case of technical data theft, the AM service provider has two sets of technical data to consider. The first set (Set A) would originate with the AM service provider; the second set (Set B) would be part of the AM digital thread but would have originated with Attack Layer 2 components. For the digital thread data set, Set B, indemnity between the parties in case of loss would most likely be governed by commercial contractual obligations and responsibilities. For Set A, the AM service provider would consider the value to business and loss damage. If the information regarded a unique item underpinning the business, the value and thus security investment incentive would be high. If the loss would result in a significant market share decrease or loss of competitive advantage, the security investment incentive would be high.

While the technical data in and of itself might not have an initial high value to the manufacturer, it has higher value when considered as a preliminary step for either a sabotage attack or illegal part manufacturing. In order to facilitate a stealthy, targeted AM sabotage attack damaging either the AM part, the AM equipment, or the environment utilizing the part, the attacker will need information such as part geometry, process parameters, or required mechanical properties. All of these are considered technical data for the purposes of our discussion. Arguably, therefore, the attacker will need to engage in technical data theft to achieve the sabotage target.

**FIGURE 16.**
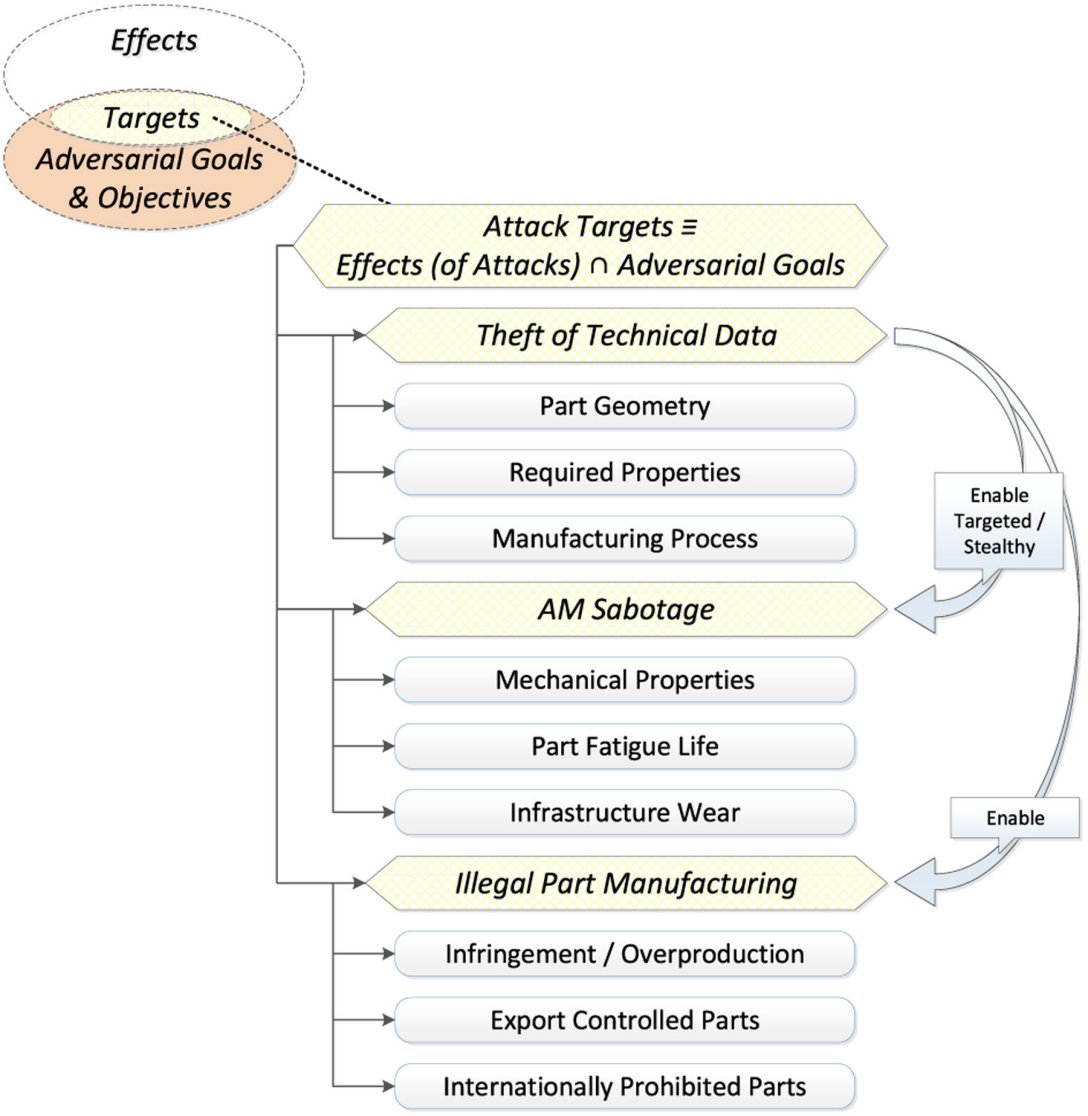
Technical data theft as preliminary target (derived from [23]).

Similarly, to engage in illegal part manufacturing by manufacturing without or exceeding authorization requires obtaining the technical data. In either instance, sabotage or illegal manufacturing, technical data theft becomes a preliminary, enabling step thereby raising the security value above the initial data set value and increasing the security investment incentive. Technical data theft as the initial target and as a required, precursor step for sabotage and illegal part manufacturing is summarized in Fig. 16.

At a high abstraction level, there appears to be significant overlap with regard to technical data theft liability between AM and SM. The complexity and need for different evaluations arise in the concentrated technical data intrinsic to AM, the uninterrupted AM digital thread from design to finished product, and the potential for the AM data owner to act as an unknowing facilitator for sabotage attacks or illegal part manufacturing. With such complexity and integration, the AM service provider will have to weight the cost-benefit analysis differently than the individual SM workflow components do.

##### b: SABOTAGE ATTACK

When contemplating security measures to address sabotage attacks, the AM service provider will consider the sabotage target. The target could be the AM part, the system in which the part is deployed such as demonstrated in *dr0wned* [35], or the AM equipment, a feasible possibility illustrated by the equipment attack in Stuxnet [30]. Also of concern when analyzing the cost-benefit and liability exposure is the injured party. Conceivably the damages could be limited to the AM service provider in the part or equipment sabotage scenarios. However, damage to the deployed system could have farther reaching damage.

Consider the recent advances in additively manufactured turbine blades. Siemens has successfully demonstrated additively manufactured power turbine blades [77] and GE has incorporated additively manufactured blades in the GE9X jet engine designed for Boeing 777X jets [78]. However, traditionally manufactured blades have been suspected of failing in jet engines due to metal fatigue in several inflight incidents, two of which resulted in fatalities [79]–[83].

Additionally, premature part fatigue and reduced strength have been studied in AM cyber-security literature [35], [41], [49]. Therefore, it is not unreasonable to envision a scenario in which a malicious actor attempts to sabotage an additively manufactured blade in order to cause airplane jet engine failure, possibly resulting in fatalities. Even without physical injuries, airlines could suffer substantial immediate economic damage if the Federal Aviation Administration (FAA) grounded similar planes. Further, the companies involved in the AM digital thread could suffer a loss of reputation and the public could lose confidence in additively manufactured parts in general. Prior evaluation and implementation (or lack thereof) of cyber-security measures would be considered when assessing liability damages following an incident [65], [84].

While sabotage attacks are also possible in SM, the cost-benefit analysis is different for AM as compared to SM. This is partially due to the difficulty in detecting introduced flaws in AM parts. Other contributing factors are the higher exposure of AM service providers to potentially malicious actors, the higher ROI for an adversary in AM than in SM, and the anticipated growth in AM due to new functionalities, design opportunities, and the expected transition from SM to AM technology envisioned by numerous manufacturing companies.

##### c: ILLEGAL PART MANUFACTURING

Although illegal part manufacturing does not precisely fit the attack analysis framework presented in subsection III-B, it can still be examined along implementation decision concerns. Illegal part manufacturing includes manufacturing without authorization or exceeding prior legitimate authorization. It also refers to manufacturing prohibited or export-controlled items. In this case, the external malicious actor would need to possess a manufacturing capability or have access to a manufacturing environment. Therefore, their target can be considered the technical data required to produce the item. For the AM service provider weighing whether to implement security measures to address this threat, the security considerations are essentially those of technical data theft, including loss of profit, market share, and advantage. There is an added potential exposure to international or criminal sanctions in the case of prohibited or export-controlled items if the failure to protect the data is such that it could be deemed to have facilitated the illegal part manufacturing.

As this area and potential for harm continues to grow and receive additional exposure in the AM security research, AM service providers will need to consider implementing restrictions and safeguards that will provide technical means for defending against the inevitable circumvention attacks. The cost-benefit and liability exposure analysis for AM service providers differs from SM in this area in that the current SM restrictions and safeguards are insufficient to address these issues and the potential for using an AM service provider for illegal manufacturing appears limitless.

#### 2) ATTACK LAYER 2

Attack Layer 2 consists of those workflow components recognized as supply chain. This level includes the AM equipment manufacturer, part designer, and feedstock and power suppliers. It does not include the post-production transportation depicted in Fig. 2. When considering which security measures to implement, the AM supplier evaluates all security threat categories.

##### a: TECHNICAL DATA THEFT

At the supply chain level, the technical data would again be valuable to the AM supplier as data owner. It is also possible that the AM supplier would possess part of the AM service provider’s technical data in the form of stated specifications requirements. The same Attack Layer 1 analyses concerning business value and lost market share or advantage would apply. Similarly, indemnity between the parties would again be governed by commercial contractual obligations and responsibilities.

Unique to this level is the need to consider that the technical data theft at this layer could be used to initiate a cross layer attack against the AM service provider – the enabling technical data theft depicted in Fig. 16 is not restricted to the same attack layer because a Layer 2 theft can be used to facilitate a Layer 1 sabotage. However, the AM supplier may consider the threat remote and rely on the AM service provider implementing their own preventative security measures. The difficulty with this rationale is that the Layer 2 theft might have the effect of transforming the actor from malicious outsider into a malicious insider for the cross layer attack.

While there is overlap between AM and SM with regards to technical data theft, there are differences due to the integration of process parameters into the AM data set and the integration of the uninterrupted AM digital thread. In comparison, in SM the data for each component’s requirements can remain with that component. For example, the SM material supplier does not need to send to the SM part manufacturer how the source material was produced. That information is not necessary for the SM CNC machines to operate successfully.

The AM environment creates a complex security interdependency. The AM workflow elements at Layer 2 have the typical cost-benefit and liability analyses where they consider data value and security costs as to themselves; however, they also need to consider that they are also in essence providing security for the other Layer 2 and Layer 1 workflow participants. However, as in SM, the perception of security benefit as opposed to fixed security costs remains low the further removed from the target and the incentive still decreases with distance. But in AM, each component can arguably be considered a proxy for the security of the whole workflow, a factor which should affect the AM cost-benefit weighting differently than in SM where components can consider segregation as a security measure in their cost-benefit analysis.

##### b: SABOTAGE ATTACK

For AM, the sabotage attack at this level would most likely be a cross layer attack where the supply chain commodities were modified in order to damage the part or equipment at the manufacturing level or the deployed system at the end user level. An example would be manipulating feedstock characteristics which might cause part failure due to poor quality or might cause equipment damage due to a chemical interaction explosion.

Attacks originating from a Layer 2 source have the tendency to affect numerous manufactured parts at once. Targeting attacks originating from a Layer 2 source are rather difficult given the indiscriminate nature of the supply chain and distance from the end user. With the distance, the damage would appear to be a remote risk to the AM supplier and the AM service provider security would be an intervening factor between supplier security measures and ultimate damage.

With the perception of high cost for low benefit, the AM supplier would most likely assign a lower priority for sabotage attack-related measures as opposed to technical data theft. However, as discussed in technical data theft above, the AM component becomes a proxy for the security of the workflow due to the integrated and complex nature of the AM workflow. This changes the cost-benefit and liability exposure analysis for the AM component to require including a distant benefit as compared to SM where the component has some ability to rely on segregation and shorter digital threads which do not span the entire workflow.

##### c: ILLEGAL PART MANUFACTURING

As with Attack Layer 1, illegal part manufacturing becomes primarily a technical data theft threat in that the external malicious actor would need access to a manufacturing environment to accomplish the goal of unauthorized or prohibited item manufacturing. The AM supplier is unlikely to highly prioritize measures identified as specifically preventing illegal part manufacturing. However, given the apparently unlimited growth potential in illegal part manufacturing and the potential for global harm, it is necessary to develop safeguards and restrictions since the current SM measures are insufficient. Responsibility for the cost of development, deployment, and then defense against circumvention attacks will become an AM industry issue as the theoretical harm evolves toward reality.

## VI. AM VERSUS SM SECURITY SYNOPSIS

In this paper, we analyzed Additive Manufacturing (AM) security from three awareness perspectives — exposure to an attack, evaluation, and liability exposure. Throughout our analysis, we identified aspects that are characteristic for AM, even when compared to the closely-related Subtractive Manufacturing (SM). We limited the scope of our comparison to SM, reserving comparisons to other manufacturing technologies to future research.

**FIGURE 17.**
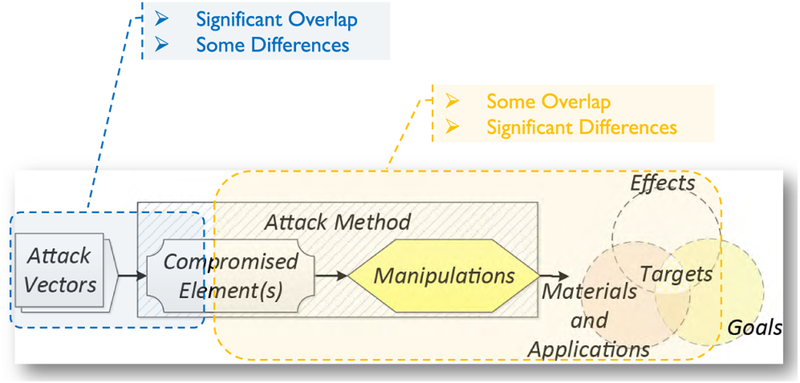
Overlap and differences between AM and SM securities (derived from [23]).

For the exposure perspective, we structured our analysis according to the AM attack analysis framework introduced by Yampolskiy *et al*. [23]. Our analysis has shown that, while there is a significant overlap between AM and SM security, numerous aspects are unique and require a dedicated consideration by security experts. See Fig. 17 for a graphical depiction of the overlap areas.

The attack analysis framework begins with attack vectors. The ways in which attack vectors can be used to compromise manufacturing workflow elements are where the majority of the identified similarities between the AM and SM security exist. However, subtle distinctions originate from the differences in the manufacturing environments. Among the environmental factors, AM has higher exposure to potentially malicious actors, increased potential for fraudulent behavior by participant actors, and easier theft due to concentrated technical data. This leads to a substantially higher Return on Investment (ROI) for an adversary in AM than in SM. Moving further right in the attack analysis framework to manipulations, we identified that substantial differences exist between AM and SM. For example, in sabotage attacks, a variety of manipulations can be used to introduce hard-to-detect, part-tailored internal defects that could cause system failures as well as failures optimized for the operational conditions unique to an additively manufactured part. While defects that reduce tensile strength or other parameters are also possible in SM, they are limited to external defects for which detection with non-destructive evaluation (NDE) methods is rather trivial.

For the evaluation perspective, we structured our analysis based upon the Risk Management Framework (RMF). While in the exposure perspective, the similarities between the AM and SM technologies arise at the beginning of the process, in the RMF process it is the differences which become apparent in the beginning. The differences arise from the complex, integrated and shifting AM workflow which makes describing the “system” and defining system boundaries for the *Prepare* and *Categorize* steps problematic. The potentially shifting nature of roles in AM also can result in necessary yet costly reassessments in comparison to the relatively stable SM workflow.

For the liability exposure perspective, we structured our analysis through extending the sabotage attack liability analysis framework introduced by Graves *et al*. [65]. The analysis framework separates the AM workflow into two layers. The layers distinguish between manufacturers and suppliers, distance from injury, and targeting ability and attack scope. At each layer, we identified the cost-benefit and liability exposure considerations for each of the three AM security threats of technical data theft, sabotage attacks, and illegal part manufacturing. The impact of fixed security costs and perceived security benefit, as well as the impact of potential liability exposure, on security implementation decision making was more nuanced for AM due to the integrated nature of the digital thread and workflow environment.

Each of the analysis discussions highlighted distinct differentiations between AM and SM security concerns which necessitate research and treatment apart from that of SM.

## VII. CONCLUSION

Additive Manufacturing (AM) is demonstrating the potential to become a mainstream manufacturing technology in the near future with a growing industry penetration around the world. As additively manufactured parts will be used in a wide range of applications, including but not limited to safety-critical parts in defense and aerospace systems, the security of the underlying AM technology is extremely important.

In this paper, we analyzed AM Security from three awareness perspectives — exposure to an attack, system security evaluation, and the implementation weighting of security cost-benefits and potential liability. For each of these perspectives, we both identified aspects that are characteristic for AM and highlighted which of those aspects are distinct from the closely related Subtractive Manufacturing (SM) using Computer Numerical Controlled (CNC) machines. Our analysis shows that it would be wrong to assume that AM is secure if the security posture used in SM is inherited and applied to AM as is. While we identified overlap between AM and SM security, AM requires a separate and unique perspective provided by experts with relevant domain expertise.

## Supplementary Material

Sup1

## Figures and Tables

**TABLE 1.** Mnemonic reference mapping.

	Mnemonic-Citation Mapping
Mnemonic	Reference
belikovetsky2016dr0wned	S. Belikovetsky, M. Yampolskiy, J. Toh, and Y. Elovici, “drOwned - Cyberphysical attack with additive manufacturing,” 2016.
belikovetsky2017dr0wned	S. Belikovetsky, M. Yampolskiy, J. Toh, J. Gatlin, and Y. Elovici, “drOwned - Cyber-physical attack with additive manufacturing,” in 11th USENIX Workshop on Offensive Technologies (WOOT 17). Vancouver, BC: USENIX Association, 2017, p. 16. [Online]. Available: https://www.usenix.org/conference/wootl7/workshopprogram/presentation/belikovetsky
do2016data	Q. Do, B. Martini, and K. K. R. Choo, “A data exflltration and remote exploitation attack on consumer 3d printers,” IEEE Transactions on Information Forensics and Security, vol. 11, no. 10, pp. 2174–2186, 2016.
eset2012acadmedrea	ESET, “ACAD/Medre.A: 10000’s of AutoCAD Designs Leaked in Suspected Industrial Espionage,” 2012, white paper. [Online]. Available: https://www.welivesecurity.corn/media_files/white-papersZESET_ACAD_Medre_A_whitepaper.pdf
faraque2016acoustic	M. A. AlFaruque, S. R. Chhetri, A. Canedo, and J. Wan,”Acoustic side-channel attacks on additive manufacturing systems,” in 2016 ACM/IEEE 7th International Conference on Cyber-Physical Systems (ICCPS). IEEE, 2016, pp. 1–10.
hojjati20161eave	A. Hojjati, A. Adhikari, K. Struckmann, E. Chou, T. N. Tho Nguyen, K. Madan, M. S. Winslett, C. A. Gunter, and W. P. King, “Leave your phone at the door: Side channels that reveal factory floor secrets,” in Proceedings of the 2016 ACM SIGSAC Conference on Computer and Communications Security. New York, NY, USA: ACM, 2016, pp. 883–894.
ilie2017in-built	A. Hie, H. Ali, K. Mumtaz, In-built customised mechanical failure of 3161 components fabricated using selective laser melting, Technologies 5 (1) (2017) 9.
moore2017implications	S. B. Moore, W. B. Glisson, and M. Yampolskiy, “Implications of malicious 3d printer firmware,” in Proceedings of the 50th Hawaii International Conference on System Sciences. IEEE, 2017, pp. 6089–6098.
moore2016vulnerability	S. Moore, P. Armstrong, T. McDonald, M. Yampolskiy, “Vulnerability analysis of desktop 3d printer software,” Resilience Week (RWS), IEEE, 2016, pp. 46–51.
pope2016hazard	G. Pope and M. Yampolskiy, “A Hazard Analysis Technique for Additive Manufacturing,” in Better Software East Conference, 2016, p. 17. [Online]. Available: http://arxiv.org/abs/1706.00497
slaughter2017how	A. Slaughter, M. Yampolskiy, M. Matthews, W. E. King, G. Guss, and Y. Elovici, “How to ensure bad quality in metal additive manufacturing: In-situ infrared thermography from the security perspective,” in Proceedings of the 12th International Conference on Availability, Reliability and Security. New York, NY, USA: ACM, 2017, pp. 78:1–78:10.
song2016my	C. Song, F. Lin, Z. Ba, K. Ren, C. Zhou, and W. Xu, “My smartphone knows what you print: Exploring smartphone-based side-channel attacks against 3d printers,” in Proceedings of the 2016 ACM SIGSAC Conference on Computer and Communications Security. NewYork,NY,USA:ACM, 2016, pp. 895–907.
sturm2014cyber-physical	L. Sturm, C. Williams, J. Camelio, J. White, and R. Parker, “Cyber-physical vulnerabilities in additive manufacturing systems,” Context, vol. 7, p. 8, 2014.
xiao2013security	Xiao Zi Hang (Claud Xiao), “Security attack to 3d printing,” 2013, keynote at XCon2013. [Online]. Available: http://www.claudxiao.net/Attack3DPrinting-Claud-en.pdf
yampolskiy2017evaluation	M. Yampolskiy, W. King, G. Pope, S. Belikovetsky, and Y. Elovici, “Evaluation of additive and subtractive manufacturing from the security perspective,” in International Conference on Critical Infrastructure Protection. Springer, 2017, pp. 23–44.
yampolskiy2014intellectual	M. Yampolskiy, T. R. Andel, J. T. McDonald, W. B. Glisson, and A. Yasinsac, “Intellectual property protection in additive layer manufacturing: Requirements for secure outsourcing,” in Proceedings of the 4th Program Protection and Reverse Engineering Workshop. New York, NY, USA: ACM, 2014, p. 7.
yampolskiy2015security	M. Yampolskiy, L. Schutzle, U. Vaidya, and A. Yasinsac, “Security challenges of additive manufacturing with metals and alloys,” in Critical Infrastructure Protection IX. Springer, 2015, pp. 169–183.
yampolskiy2018security	M. Yampolskiy, W. E. King, J. Gatlin, S. Belikovetsky, A. Brown, A. Skjellum, and Y. Elovici, “Security of Additive Manufacturing: Attack Taxonomy and Survey,” Additive Manufacturing, vol. 21, pp. 431–457, 2018.
yampolskiy2016using	M. Yampolskiy, A. Skjellum, M. Kretzschmar, R. A. Overfelt, K. R. Sloan, and A. Yasinsac, “Using 3D Printers as Weapons,” International Journal of Critical Infrastructure Protection, vol. 14, pp. 58–71, 2016.
zeltmann2016manufacturing	S. E. Zeltmann, N. Gupta, N. G. Tsoutsos, M. Maniatakos, J. Rajendran, and R. Karri, “Manufacturing and security challenges in 3d printing,” Jom, vol. 68, no. 7, pp. 1872–1881, 2016.

**TABLE 2.** Security implications of AM environment.

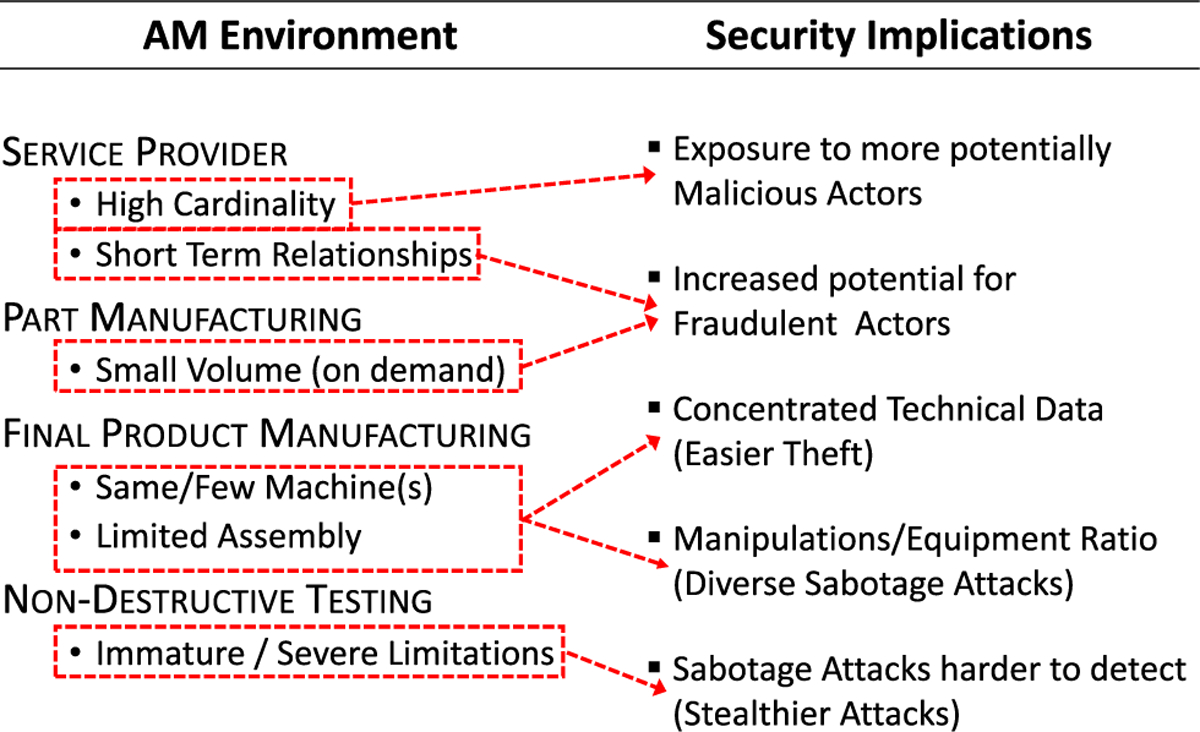

**TABLE 3.** Compromisable AM workflow elements.

Cyber Components
Design Files and Specifications
Process Modeling and Optimization Software (topology optimization, distortion correction, etc.)
Sensor Data Feedback
Physical Commodities
Feedstock (powder, inert gas, etc.)
Power Grid
Cyber-Physical Components
Actuator Signals/Commands
Sensor Data
Workflow Steps
Feedstock Characterization
Powder Recycling
Post-Processing

**TABLE 4.** Attack methods for technical data theft.

	AM	SM
Cyber Attacks		
Spear Phishing	✓	✓
- belikovetsky2016dr0wned		
- belikovetsky2017dr0wned		
Network Session Hijacking	✓	✓
- do2016data		
Worms: ACAD/Medre.A	✓	✓
- eset2012acad		
Cyber-Physical Attacks		
Side Channels	✓	✓
- yampolskiy2014intellectual		
Side Channels (Acoustic)	✓	✓
- faruque2016acoustic		
Side Channels (Acoustic and Electro-Magnetic)	✓	✓
- song2016my		
- hojjati20161eave		

✓ - possible,

(✓) - Conditionally Possible,

✗ - Not Possible

**TABLE 5.** Attack methods for sabotage.

	AM	SM
Direct Attacks		
External Shape: Dimensions	✓	✓
- xiao2013security		
- sturm2014cyber-physical		
External Shape: Part Geometry	✓	✓
- moore2017implications		
Internal Cavities: Voids	✓	✗
- sturm2014cyber-physical		
- belikovetsky2016dr0wned		
- belikovetsky2017dr0wned		
Internal Cavities: Source Material Substitution	✓	✗
- zeltmann2016manufacturing		
Build Direction	✓	✗
- yampolskiy2015security		
- zeltmann2016manufacturing		
Process Control Parameters	✓	✗
- yampolskiy2015security		
Compromised Equipment: Communication Timing	✓	✗
-pope2016hazard		
- yampolskiy2017evaluation		
Compromised Equipment: Power Supply Characteristics	✓	✗
-pope2016hazard		
- yampolskiy2017evaluation		
State Estimation Attacks		
Process Sensor Information: IR Thermography	✓	✗
- slaughter2017how		

✓ - possible,

(✓) - Conditionally Possible,

✗ - Not Possible

**TABLE 6.** Attack targets for technical data theft.

	AM	SM
Part Specification: Part Geometry	✓	✓
- belikovetsky2016dr0wned		
- belikovetsky2017dr0wned		
- do2016data		
- faruque2016acoustic		
- song2016my		
- hojjati20161eave		
Part Specification: Required Properties	✓	✗
- yampolskiy2014intellectual		
Manufacturing Process Specification	✓	✗
- yampolskiy2014intellectual		
Post-Processing Specification	✓	(✓)
- yampolskiy2018security		
Indirect Manufacturing	✓	(✓)
- yampolskiy2018security		

✓ - possible,

(✓) - Conditionally Possible,

✗ - Not Possible

**TABLE 7.** Attack targets for sabotage.

	AM	SM
Manufactured Part		
Function: Mechanical Properties:Tensile Strength	✓	(✓)
- sturm2014cyber-physical		
- zeltmann2016manufacturing		
Function: Mechanical Properties:Material Fatigue	✓	(✓)
- belikovetsky2016dr0wned		
- belikovetsky2017dr0wned		
Equipment		
Mechanical Components: Increased Wear	✓	✓
- yampolskiy2016using		
Environment of Manufactured Part		
Device Properties: Life Time	✓	✓
- belikovetsky2016dr0wned		
- belikovetsky2017dr0wned		
Environment of Equipment		
Machine Shop Tools: Physical Damage	✓	✗
- yampolskiy2016using		

✓ - possible,

(✓) - Conditionally Possible,

✗ - Not Possible

**TABLE 8.** *Prepare* tasks and outcomes analyzed for AM [21].

Task	Outcome	Cybersecurity Framework
P-9: System Stakeholders	The stakeholders having an interest in the system are identified	ID.AM Asset Management ID.BE Business Environment
P-10: Asset Identification	Stakeholder assets are identified	ID.AM Asset Management
P-11: Authorization Boundary	The authorization boundary (i.e., system) is determined	
P-12: Information Types	The types of information processed, stored, and transmitted by the system are identified	ID.AM-5 Asset Management
P-13: Information Life Cycle	All stages of the information life cycle are identified and understood for each information type processed, stored, or transmitted by the system	ID.AM Asset Management
P-14: Risk Assessment	A system-level risk assessment is completed or an existing risk assessment is updated	ID.RA Risk Assessment ID.SC-2 Supply Chain Risk Management
P-16: Enterprise Architecture	The placement of the system within the enterprise architecture is determined	

**TABLE 9.** *Categorize* tasks and outcomes analyzed for AM [21].

Task	Outcome	Cybersecurity Framework
C-l: System Description	The characteristics of the system are described and documented	Profile
C-2: System Categorization	A security categorization of the system, including the information processed by the system represented by the organization-identified information types, is completed	ID.AM Asset Management
	Security categorization results are documented in the security, privacy, and supply chain risk management plans	Profile
	Security categorization results are consistent with the enterprise architecture and commitment to protecting organizational missions, business functions, and mission/business processes	Profile
	Security categorization results reflect the organization’s risk management strategy	

## Footnotes

1 For this paper, AM specifically refers to industrial- or commercial-level additive manufacturing and not hobby or consumer-grade 3D printers.

2 For this paper, use of the term SM implies SM using CNC machines.

3 The American Society for Testing and Materials (ASTM) defines seven different AM process categories [24], [25]; each of these categories can have several sub-categories, often referred to as AM technology.

4 As of May 2019, the *3D Printing Business Directory* website [26] lists 783 companies offering 3D-printing service, 117 of which provide metal printing.

5 Many functional and safety-critical AM parts are produced in metal.

6 It should be noted that this was used only as a preparation stage for the follow-up sabotage attack.

7 In this context, anisotropic means that mechanical properties vary according to build direction.

8 In Powder Bed Fusion (PBF), scanning strategy determines the pattern in which the source material is melted by the heat source.

9 Manufacturing is one of the 16 Critical Infrastructure Sectors identified in Presidential Policy Directive 21 (PPD-21) [60]. The Department of Homeland Security (DHS) includes Primary Metals Manufacturing and Machinery Manufacturing as core sector industries [61].

10 This system boundary assumes a direct connection between the devices. In AM operational settings, the devices are often geographically separated and connected through network devices. Those network devices could be considered as part of the system or provided as a service which would trigger additional security concerns and measures such as Service Level Agreements (SLAs) which are not considered in our scenario.

11 For our analysis, the “*controlled environment*” of Section III and the “*environment of operation*” of this section share the same boundary and workflow components. We note that may not always be the case.

12 A real-world example is the Target breach where a third-party heating, ventilation, and air conditioning vendor compromised through a phishing attack provided access to the retailer, resulting in data theft. [75], [76]
