# Supplementary figures and images for: Characteristic Aspects of Additive Manufacturing Security From Security Awareness Perspectives
Source: IEEE Access. Author manuscript; Available in PMC 2020 Feb 28. (PMC7047715; doi:10.1109/access.2019.2931738)

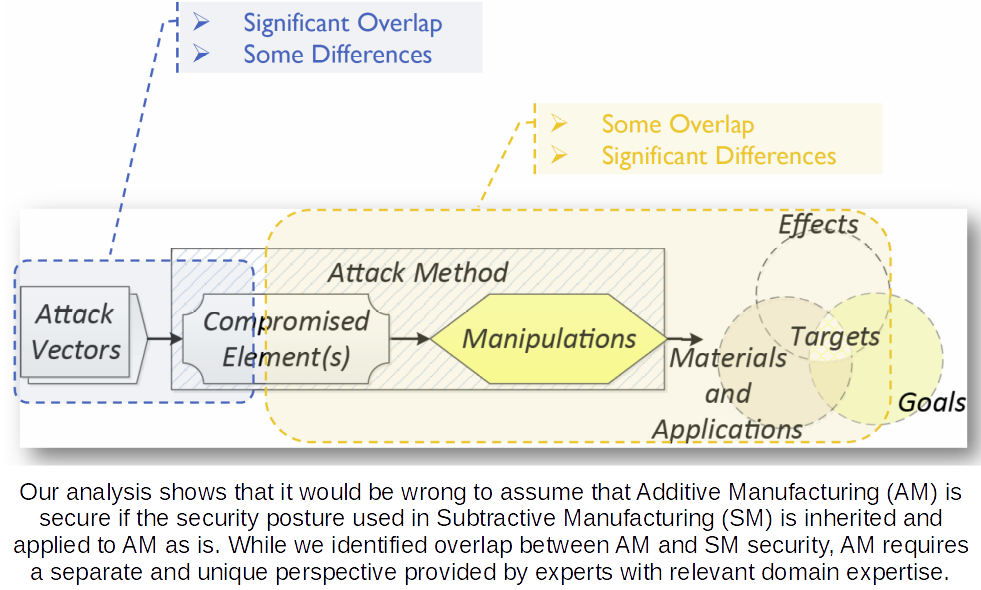

Supplement: Sup1 [file NIHMS1540316-supplement-Sup1.png]
